# Screening Central Asian Wheat Germplasm for Powdery Mildew Resistance and Molecular Characterization of Disease Resistance Genes

**DOI:** 10.3390/plants15203106

**Published:** 2026-10-10

**Authors:** Muhammad Nosherwan, Muhammad Hammad Latif, Junyu Ma, Tao Chen, Muhammad Saqlain, Qianyi Guo, Ke Zhang, Chunxiao Wang, Wei Zhu, Lili Xu, Dandan Wu, Yonghong Zhou, Houyang Kang, Yinghui Li

**Affiliations:** 1 State Key Laboratory of Crop Gene Exploration and Utilization in Southwest China, Sichuan Agricultural University, Chengdu 611130, China; nosherwan320@gmail.com (M.N.); hammadlatif281@gmail.com (M.H.L.); 15964155093@163.com (J.M.); caz715610@163.com (T.C.); 2024512001@stu.sicau.edu.cn (M.S.); 18227145123@163.com (Q.G.); m15538341372@163.com (K.Z.); chunxiao202610@163.com (C.W.); zhuwei202209@163.com (W.Z.); xulili_0627@126.com (L.X.); wudandan@sicau.edu.cn (D.W.); zhouyh@sicau.edu.cn (Y.Z.); houyang.kang@sicau.edu.cn (H.K.); 2 Triticeae Research Institute, Sichuan Agricultural University, Chengdu 611130, China

**Keywords:** wheat, powdery mildew, resistance identification, molecular marker, resistance breeding

## Abstract

Powdery mildew, caused by *Blumeria graminis* f. sp. *tritici* (*Bgt*), is a widespread and destructive wheat disease. Employing host powdery mildew (*Pm*) resistance genes is the most cost-effective and environmentally sustainable approach to managing this disease. In this study, we evaluated seedling resistance in 192 wheat germplasm accessions from three Central Asian countries, Kazakhstan, Kyrgyzstan, and Tajikistan, and identified five accessions with high resistance against *Bgt* isolate E09. Testing with 12 additional *Bgt* isolates demonstrated that these five accessions may contain broad-spectrum resistance. Molecular analysis using functional *Pm* resistance gene markers revealed that two durum wheat accessions, ZY110 and ZY117, may carry *Pm68*, and one common wheat accession, ZY1, may carry a candidate *Pm26* allelic variant. Notably, none of the tested known *Pm* gene markers were detected in ZY47 and ZY95. Morphological and GISH analysis confirmed that ZY47 and ZY95 possess the rye genome (AABBRR). Molecular marker analysis of three F_2_ segregating populations (ZY110 × Kronos, ZY117 × Langdon, and ZY1 × Chuanmai 42) confirmed that each contained a single resistance gene; the diagnostic markers for *Pm68* co-segregated with *Bgt* resistance, while the marker genotypes for *Pm26* co-segregated with the expected recessive inheritance model. Sequence alignment confirmed the full-length sequence of *Pm68* in durum wheat accessions ZY110 and ZY117, supporting that *Pm68* has been used in Central Asian wheat breeding. The candidate *Pm26* allelic variant in ZY1, and the candidate sources of novel rye-derived powdery mildew resistance in accessions ZY47 and ZY95, may enrich the powdery mildew resistance gene pool for wheat breeding.

## 1. Introduction

Common wheat *(Triticum aestivum* L., 2n = 6x = 42, AABBDD) is a major staple crop worldwide, contributing significantly to both human caloric intake and the supply of essential nutrients [1]. Powdery mildew, caused by the biotrophic fungus *Blumeria graminis* f. sp. *tritici* (*Bgt*), is considered one of the most damaging foliar diseases globally [2]. Deployment of resistance (R) genes in the host plant represents the most economical strategy for conferring genetic resistance to this disease [3]. Currently, 72 officially designated powdery mildew (*Pm*) resistance genes (*Pm1*–*Pm72*), along with more than 140 functional alleles, have been documented in wheat and its wild relatives. To date, around 25 *Pm* resistance genes have been cloned, most of which encode nucleotide-binding and leucine-rich repeat receptor (NLR)-class proteins, including singleton NLRs (*Pm1a*, *Pm2*, *Pm3b*/*Pm8*/*Pm17*, *Pm12*/*Pm21*, *Pm41*, *Pm55*, *Pm60/MlIW172*/*MlWE18*, *Pm69, PmAeu1*, *PmTR1*/*PmTR3*, *Pm37*/*PmNCA6*, and *PmD479*); paired NLRs (*RXL-Pm5e*, *Pm26*/*TdCNL1-TdCNL5*, and *Pm68*/*PmW183*/*MlIW39*); and NLRs with integrated domains (IDs) (*Pm6Sl* with a BED domain, *Pm52*/*Pm6* with a kinase domain). *Pm24* and *WTK4* are tandem kinase proteins (TKPs), whereas *Pm36* and *Pm57* represent TKP-IDs fused to a transmembrane domain and a von Willebrand factor A domain, respectively. In addition, *Pm4* and *Pm13* encode kinases with IDs (*Pm4* with a transmembrane domain and *Pm13* with a mixed lineage kinase-like domain), while *Pm38* and *Pm46* function as transporters (an ABC transporter and a hexose transporter, respectively) [4,5,6,7,8].

Moreover, due to pathogen evolution, extensively utilized genes in wheat resistance breeding such as *Pm1–Pm6* and *Pm8* have lost effectiveness against prevalent *Bgt* isolates in some wheat-growing regions [3]. Recent studies have reported *Pm1a* virulence among *Bgt* isolates from Egypt, Israel, Turkey, and Brazil, and have also shown that *Pm1a* has lost effectiveness at both seedling and adult stages in different regions of Western Australia [9,10,11]. *Pm2* is widely used in wheat breeding; of 641 accessions screened, 33 carried the *Pm2a* haplotype, and other alleles, such as *Pm2d–2j*, represent valuable resources for breeding [3]. However, *Pm2* alleles confer race-specific resistance, while pyramiding *Pm2* and *Pm6* appears to be a valuable approach for wheat resistance breeding [12,13]. Similarly, *Pm4* alleles exhibit race-specific resistance. For instance, *Pm4b* has lost resistance in Western Australia, whereas *Pm4a* remains effective [9]. *Pm8*, introduced via the 1BL.1RS translocation, has been widely used globally since the 1960s for its agronomic value, despite its resistance breakdown across regions including China [14,15]. *Pm21* confers strong resistance against diverse *Bgt* isolates across China; however, despite being the most effective gene widely used in wheat breeding, *Pm21* is under strong selection pressure and is at risk of resistance breakdown [16]. Therefore, newly developed accessions and elite breeding lines should be evaluated against prevailing *Bgt* isolates to monitor resistance breakdown and enable the replacement of susceptible cultivars.

Common wheat (*Triticum aestivum* L., AABBDD) and durum wheat (*Triticum turgidum* L., AABB) represent the primary and secondary gene pools, respectively, whereas rye (*Secale cereale* L., RR) constitutes the tertiary gene pool, which is rich in genetic diversity for wheat breeding. Numerous *Pm* resistance genes have been identified, such as *Mld*, *Pm3h*/*Pm3d*, *PmDR147*, and *Pm68* from durum wheat [17,18,19,20]; *Pm7*, *Pm8*, *Pm17*, *Pm20*, *Pm56*, *PmTR1*/*PmTR3*, and *PmYT9* from rye [21,22,23,24,25,26,27]; *Pm5d*, *Pm5e*, *Pm24*, *Pm24b*, *Pm45, Pm47*, and *Pm61* from Chinese wheat landraces [28,29,30,31,32,33,34]; as well as *Pm59* and *Pm63* from Afghanistani and Iranian wheat landraces [35,36]. Accurate identification of resistance genes is essential for their efficient use in wheat breeding. Traditional methods are time-consuming, but advances in molecular biology have enabled marker-assisted selection using genomic DNA, allowing rapid and precise identification of resistant plants. With the increasing number of cloned resistance genes, functional markers allow direct and accurate genotyping, greatly accelerating breeding programs [37,38].

Central Asia harbors abundant and diverse wheat germplasm. However, systematic research on powdery mildew resistance has been remarkably limited in this region. In the present study, a total of 192 wheat germplasm accessions were collected from three Central Asian countries: Kazakhstan, Kyrgyzstan, and Tajikistan. We aimed to (i) screen wheat genotypes for resistance to powdery mildew; (ii) characterize the underlying *Pm* resistance genes through genetic analysis and molecular markers; (iii) sequence candidate *Pm* resistance genes to determine whether they represent known or candidate allelic variants; and (iv) evaluate the breeding potential of the resistant germplasm identified.

## 2. Materials and Methods

### 2.1. Experimental Materials

A total of 192 wheat germplasm accessions collected from three different countries in Central Asia, including 82 from Kyrgyzstan, 73 from Tajikistan, and 37 from Kazakhstan, were used for screening wheat powdery mildew disease-resistant germplasm (Table S1). Previous studies have demonstrated that this collection possesses considerable genetic and phenotypic diversity [39]. The durum wheat cultivars Langdon and Kronos, and common wheat cultivars Chuanmai 42 (CM42), CM42*ph1b*, and Shumai 830*ph1b* (SM830*ph1b*) were used as susceptible parents in crosses with the resistant accessions to produce segregating populations for genetic evaluation and molecular marker detection of their *Pm* resistance genes [40]. The 192 wheat germplasm accessions used in this study were provided by the Triticeae Research Institute at Sichuan Agricultural University.

### 2.2. Evaluation of Powdery Mildew Resistance

The *Bgt* isolate E09, along with an additional 12 isolates collected from different regions of China, was used for inoculation (Table S2). All *Bgt* isolates were maintained on the susceptible cultivar Xuezao (XZ) in our lab. The incubation and disease assessment followed the methods of Li et al. [41] with slight modifications. In brief, the first leaf of each plant was excised into three segments and cultured in square Petri dishes containing 8 g/L agar with 50 mg/L benzimidazole (J&K Scientific, Beijing, China). The dishes were inoculated with fresh powdery mildew spores in an inoculation tower and transferred into a growth chamber under a 16 h light/8 h dark cycle at 22 ± 2 °C. Firstly, plants of each wheat accession at the one-leaf stage were inoculated with *Bgt* E09 and only accessions resistant to E09 were inoculated with the other 12 isolates. For the segregation populations, 60 seeds from ZY110 × Kronos and ZY117 × Langdon and 79 seeds from ZY1 x CM42 population were sown in trays. When the first leaf had fully expanded, the resistant and susceptible parents and their F_1_ and F_2_ progeny plants were also inoculated with the *Bgt* isolate E09 using the dusting method [42] in a growth chamber. The susceptible cultivar Xuezao (XZ) was used as a positive control for susceptibility to confirm successful inoculation. Disease reactions were evaluated at 10 days after inoculation. When XZ showed typical disease symptoms, the inoculation was considered successful, and the powdery mildew resistance of the tested wheat lines was recorded. Infection types (ITs) were scored on a 0–4 scale: 0, highly resistant (no visible infection symptoms); 1, resistant (necrotic flecks with no visible sporulation); 2, moderately resistant (small colonies with absent or sparse sporulation, diameter ≤ 1 mm); 3, moderately susceptible (moderate mycelial growth and sporulation, diameter ≥ 1 mm); and 4, highly susceptible (well-developed mycelium and abundant sporulation, diameter ≥ 1 mm).

The highly resistant accessions (IT = 0) at the seedling stage were grown for phenotyping in field trials in the experimental area of Sichuan Agricultural University. To generate natural disease pressure, susceptible spreader rows of Fielder were pre-inoculated with *Bgt* E09 at the seedling stage and were also exposed to naturally occurring *Bgt* inoculum [40]. Disease evaluations were carried out multiple times from heading to grain filling (approximately late March) [43], and infection responses were scored following the same protocol described above.

### 2.3. Genetic Analysis and BSA of Resistant Wheat Accessions

To elucidate the inheritance of powdery mildew resistance in the highly resistant accessions, both the resistant and susceptible parents, along with their respective segregating F_2_ populations, were inoculated with *Bgt* isolate E09. Additionally, for the ZY1 × CM42 population, 15 progeny plants from each F_2:3_ family were evaluated for disease response. Following phenotypic scoring, the numbers of resistant and susceptible plants were recorded, and a chi-squared (χ^2^) goodness-of-fit test was applied to analyze the segregation ratio. The deviation of the observed phenotypic data from the expected Mendelian ratios was assessed using IBM SPSS Statistics 24.0 [44,45].

Bulked segregant analysis (BSA) using 55K SNP-array genotyping was performed to identify the genomic region related to powdery mildew resistance. For BSA ZY110 × Kronos and ZY117 × Langdon F_2_ populations, leaf tissues from 15 resistant and 15 susceptible plants were collected at 10 days post-inoculation (dpi) based on phenotypic scores. Equal amounts of DNA from each plant were pooled to construct resistant and susceptible bulks. The pooled DNA samples were genotyped using the wheat 55K SNP array at Tcuni Bioscience (Chengdu, China). For SNP calling, the *Triticum turgidum* cv. Kronos and cv. Langdon reference genomes were used for the ZY110 × Kronos and ZY117 × Langdon populations, respectively. To identify differential SNPs, genotype calls from the resistant and susceptible bulks were compared using the Excel formula IF(R = S, 0, 1), where R and S represent the genotype calls of resistant and susceptible bulk. SNPs with identical calls in both bulks were assigned a value of 0 and excluded, whereas SNPs with different calls were assigned a value of 1 and retained as differential SNPs. These candidates were then filtered to retain only those with homozygous genotype calls; SNPs with heterozygous or ambiguous calls were excluded. The chromosomal distribution of the retained differential SNPs was subsequently visualized as a SNP-density plot using the SRplot online platform (https://www.bioinformatics.com.cn/srplot (accessed on 24 December 2025), with a 1 Mb window size. Genomic regions enriched for differential SNPs were considered candidate regions potentially associated with powdery mildew resistance.

### 2.4. Molecular Detection

Fresh leaves were taken during the two-leaf stage of wheat, and wheat leaf DNA was extracted using the CTAB method [46]. DNA concentration was measured using a Nano drop and diluted to 50 ng/μL with ddH_2_O. Molecular markers for cloned *Pm* resistance genes were first used to screen only the highly resistant Central Asian wheat accessions. The markers that produced positive amplifications in the resistant accessions were then used to genotype each F_2_ segregation population to confirm co-segregation with powdery mildew resistance. The primer sequences of the known *Pm* resistance genes used in this study are provided in Table S3. The PCR reaction system (20 μL) consisted of DNA template (100 ng), forward primer (final concentration of 200 nmol/L), reverse primer (final concentration of 200 nmol/L), and PCR Mix (2 × Taq Master M). PCR amplification conditions were as follows: pre-denaturation at 95 °C for 5 min; 35 cycles of 95 °C for 15 s, TM of each known marker for 15 s and 72 °C for 1 min per kb (varied according to the amplicon length, as described in Table S3) and a final extension at 72 °C for 5 min. PCR products were separated on a 1% agarose gel containing Goldview nucleic acid dye at 120 V for 20 min and visualized [47].

For each population, the total number of individuals (N), the number of resistant and susceptible individuals, and the number of recombinant individuals (those showing discordance between marker genotype and phenotype) were recorded. The recombination frequency (RF) was calculated as the number of recombinant individuals divided by the total number of F_2_ individuals. Genetic distance in centimorgans (cM) was derived using the Kosambi mapping function [44]:

*d* = 25 × *ln* [(1 + 2*RF*)/(1 − 2*RF*)]

For populations with zero recombinants, the recombination frequency was recorded as 0%, and the upper 95% confidence limit was estimated as 3/N (Hanley’s rule of three), converted to cM, where N is the total number of F_2_ individuals [48,49].

### 2.5. Cytogenetic Analysis

For chromosome preparation, root-tip cells at mitotic metaphase were used, and genomic in situ hybridization (GISH) was performed according to previously reported protocols [50,51]. For GISH, total genomic DNA extracted from rye was labeled with Atto-550-dUTP (Jena Bioscience, Jena, Germany) as the GISH probe (red signal), using the Atto NT Labeling Kit. The labeling reaction (20 μL) consisted of 6 μL of DNA template (200 ng/μL), 2 μL of 10× NT labeling buffer, 2 μL of Enzyme mix (Vazyme, Nanjing, China), 2 μL of Atto-550 NT Labeling Kit reagent, and 8 μL of PCR-grade water. The reaction was incubated at 15 °C for 90 min, followed by 65 °C for 10 min, and terminated by adding 5 μL of stop buffer (Vazyme, Nanjing, China). Labeled probes were stored at −20 °C until use. All labeling steps were performed on ice and protected from light.

For hybridization, 1 μL of the GISH probe mixture was dissolved in 9 μL of HM (hybridization mixture), denatured at 90 °C for 10 min in a dry thermostat (DH200, Hangzhou Ruicheng, Hangzhou, China), and immediately chilled on ice for 10 min. Then, 3 μL of blocking DNA (Chinese Spring genomic DNA, 4000–5000 ng/μL) was added. The hybridization mixture (13 μL) was applied to the denatured chromosome preparations on slides, covered with a coverslip, and denatured at 85 °C for 5 min. The slides were then placed in an aluminum box containing moist filter paper and incubated at 50 °C for >7 h. After incubation, the slides were washed in 2× SSC at 50 °C for 10 min, followed by sequential rinses in 75% ethanol and absolute ethanol for 1 min each. After air-drying, chromosomes were counterstained with 8 μL of 4′,6-diamidino-2-phenylindole (DAPI). Images were captured and analyzed using an Olympus BX-63 fluorescence microscope (Olympus, Tokyo, Japan) equipped with a DP-80 camera. All procedures were carried out in the dark. Specific steps were performed according to Gong et al. [52].

### 2.6. Cloning and Sequence Analysis

Total DNA was extracted from young leaves of wheat accessions carrying known *Pm* resistance gene*s* using the CTAB method. The full-length *Pm26* and *Pm68* alleles were amplified from genomic DNA using PCR with specific primers (*Pm26-R1-1*/*Pm26-R1-2*/*Pm26-R1-3*, *Pm26-R2, Pm68-R1JCFL*, and *Pm68-R2JCFL*) (Table S4). PCR was performed as follows: initial denaturation at 98 °C for 10 s; 35 cycles of denaturation at 98 °C for 10 s, annealing at 60 °C for 5 s, and extension at 68 °C for 5–10 s per kb (adjusted based on amplicon length); and a final extension at 68 °C for 5 min. The cloned products were Sanger-sequenced, and the sequences were aligned with known alleles [20,53].

### 2.7. Agronomic Trait Evaluation

During the 2025-2026 wheat cropping season, a field trial was carried out at the experimental area of Sichuan Agricultural University, Chengdu, China, to assess the agronomic traits of highly resistant wheat accessions. Fifteen seeds of each accession were sown in each 1.5 m row. Each accession was grown in five adjacent rows, while rows of different accessions were located at different positions within the same experimental field. At maturity, 5 plants per accession (n = 5) were randomly selected for measurement of agronomic traits, including plant height, tiller number, spike length, number of spikelets per spike, thousand-seed weight, seed length, and seed width. Descriptive comparisons of agronomic parameters between highly resistant accessions and the control cultivar SM830 were illustrated using GraphPad Prism 9.5.1. The data were presented as mean ± standard deviation (SD).

## 3. Results

### 3.1. Screening the Resistant Accessions from the Central Asian Wheat Germplasm

**Figure 1:**
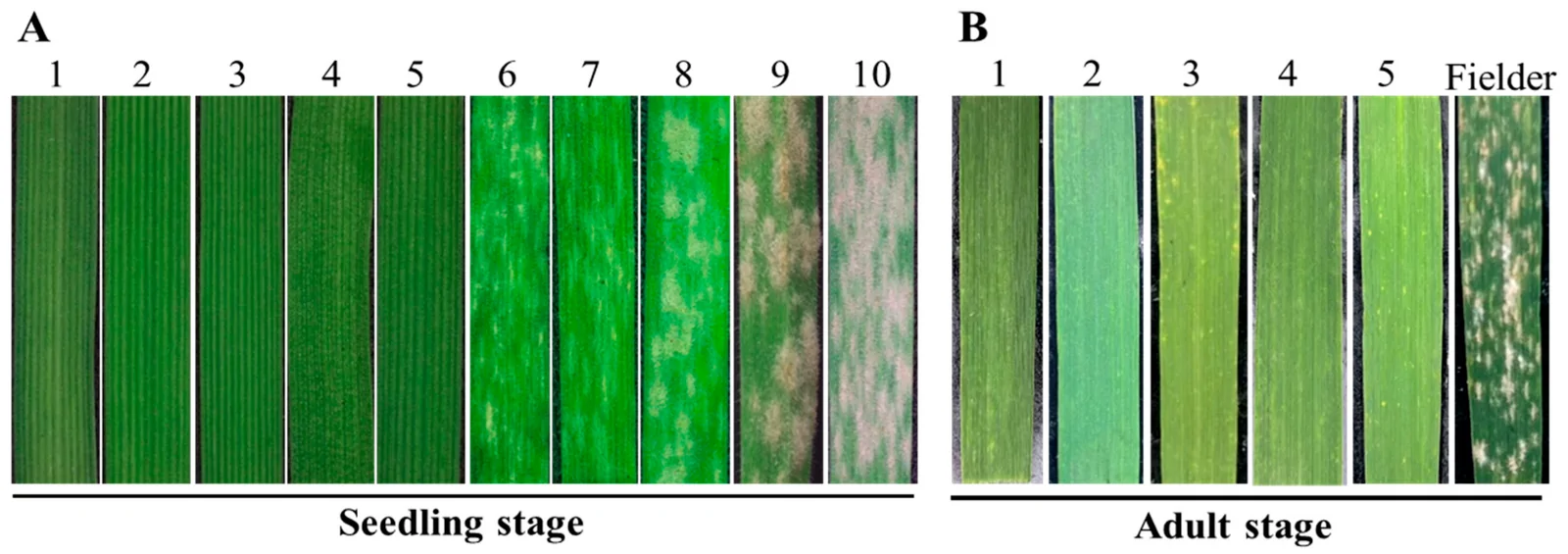
Evaluation of powdery mildew resistance in Central Asian wheat germplasm. (**A**) Seedling-stage response to *Bgt* isolate E09: 1, ZY1; 2, ZY110; 3, ZY117; 4, ZY47; 5, ZY95; 6, ZY158; 7, ZY174; 8, ZY179; 9, ZY181; 10, control (Xuezao). (**B**) Adult-plant-stage response to E09 and naturally occurring *Bgt* inoculum: 1, ZY1; 2, ZY110; 3, ZY117; 4, ZY47; 5, ZY95; 6, control (Fielder).

To initially screen for resistant wheat accessions, we inoculated 192 wheat germplasm accessions with the *Bgt* isolate E09, which is widespread in the major wheat-growing regions of North China. A wide range of infection responses was observed among the accessions, from highly resistant to highly susceptible (Table S1). About 53 out of 192 wheat germplasm accessions exhibited resistance, with ITs ranging from 0 to 2. Among these, five accessions, ZY1 (Jiyin Dong-1), ZY47 (Jiyin Dong-255), ZY110 (Tayin XM2012-11), ZY117 (Tayin XM2012-17), and ZY95 (Jiyin C-XM2012-51), were highly resistant (IT-0), while the remaining resistant accessions showed moderate resistance (IT-2), including ZY158, ZY174, and ZY179 (Figure 1A). The only five highly resistant Central Asian accessions that showed IT-0 at seedling stage against *Bgt* isolate E09 were further evaluated in the field and all maintained high resistance to powdery mildew at the adult stage under field conditions (Figure 1B).

### 3.2. Evaluation of Resistant Accessions Using Different Bgt Isolates

**Figure 2:**
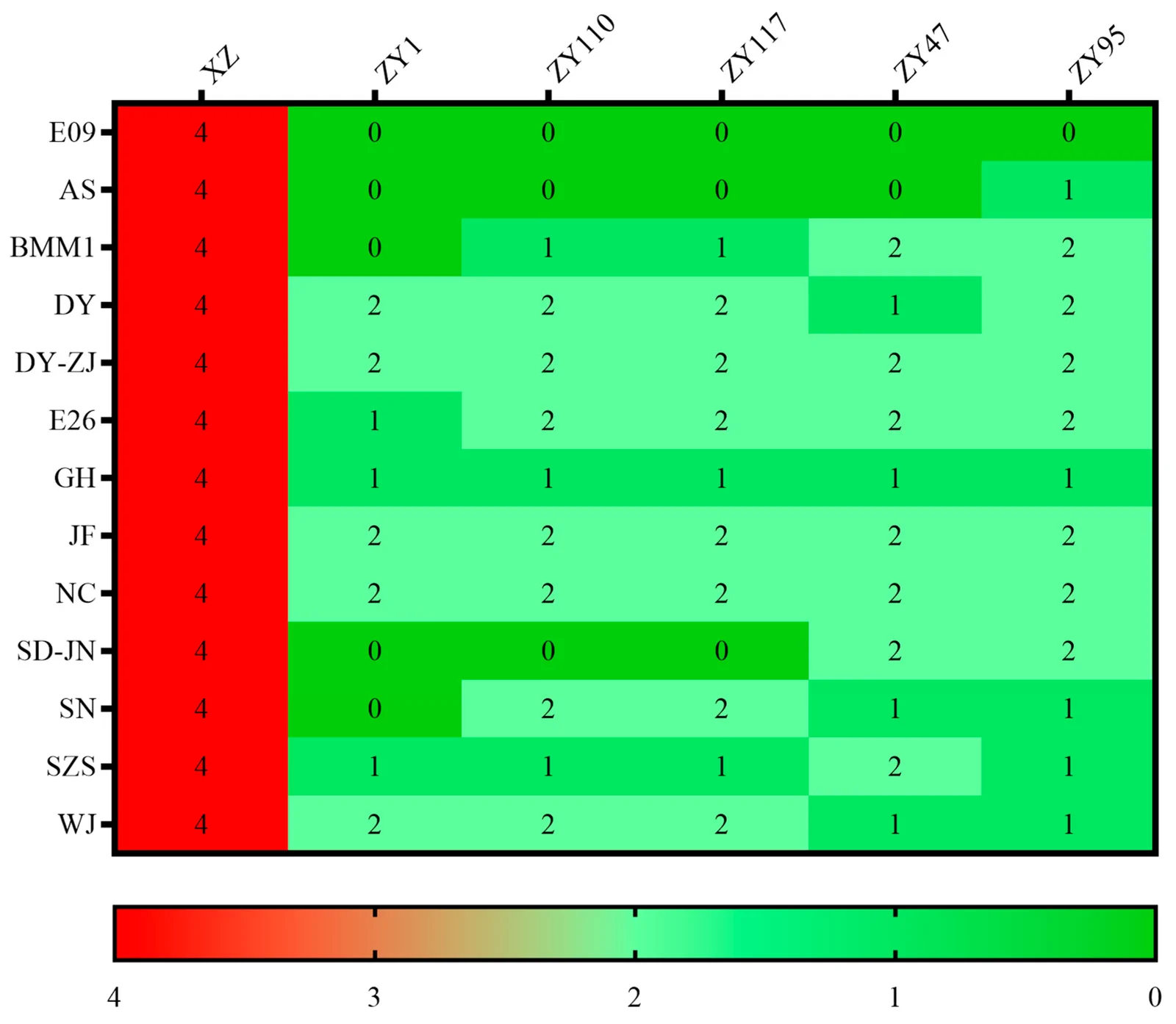
Resistance pattern of wheat accessions to thirteen *Blumeria graminis* f. sp. *tritici* (*Bgt*) strains. Infection types were scored on a 0–4 scale: 0, highly resistant; 1, resistant; 2, moderately resistant; 3, moderately susceptible; 4, highly susceptible. Note: Virulence/avirulence profile and geographical origin of 13 *Blumeria graminis* f. sp. *tritici* (*Bgt*) isolates tested against 23 *Pm* resistance genes or haplotypes in Table S2.

Subsequently, to evaluate the resistance spectrum of these five highly resistant accessions, 12 additional *Bgt* isolates were tested and Xuezao (XZ) was used as susceptible control. All five accessions showed high resistance to the majority of the isolates, with ITs of 0–2 (Figure 2). In contrast, XZ displayed a compatible interaction with all *Bgt* isolates, resulting in ITs of 4. For *Bgt* isolates E09, AS, and GH, all five accessions showed ITs of 0 or 1, characterized by either no visible signs of infection or only small necrotic flecks without detectable sporulation. For isolates DY-ZJ, JF, NC, DY, and E26, all accessions displayed moderate resistance (IT-2), except ZY47 against DY and ZY1 against E26, which showed resistance (IT-1). In these cases, a few accessions developed minor chlorotic lesions with very limited or no sporulation, still falling within the resistant category (IT ≤ 2). Overall, these results demonstrate that the five accessions possess effective resistance against the tested *Bgt* isolates, while the susceptible line serves as a reliable control for virulence assays.

### 3.3. Cytogenetic and Molecular Characterization of Wheat–Rye Amphiploid Lines

**Figure 3:**
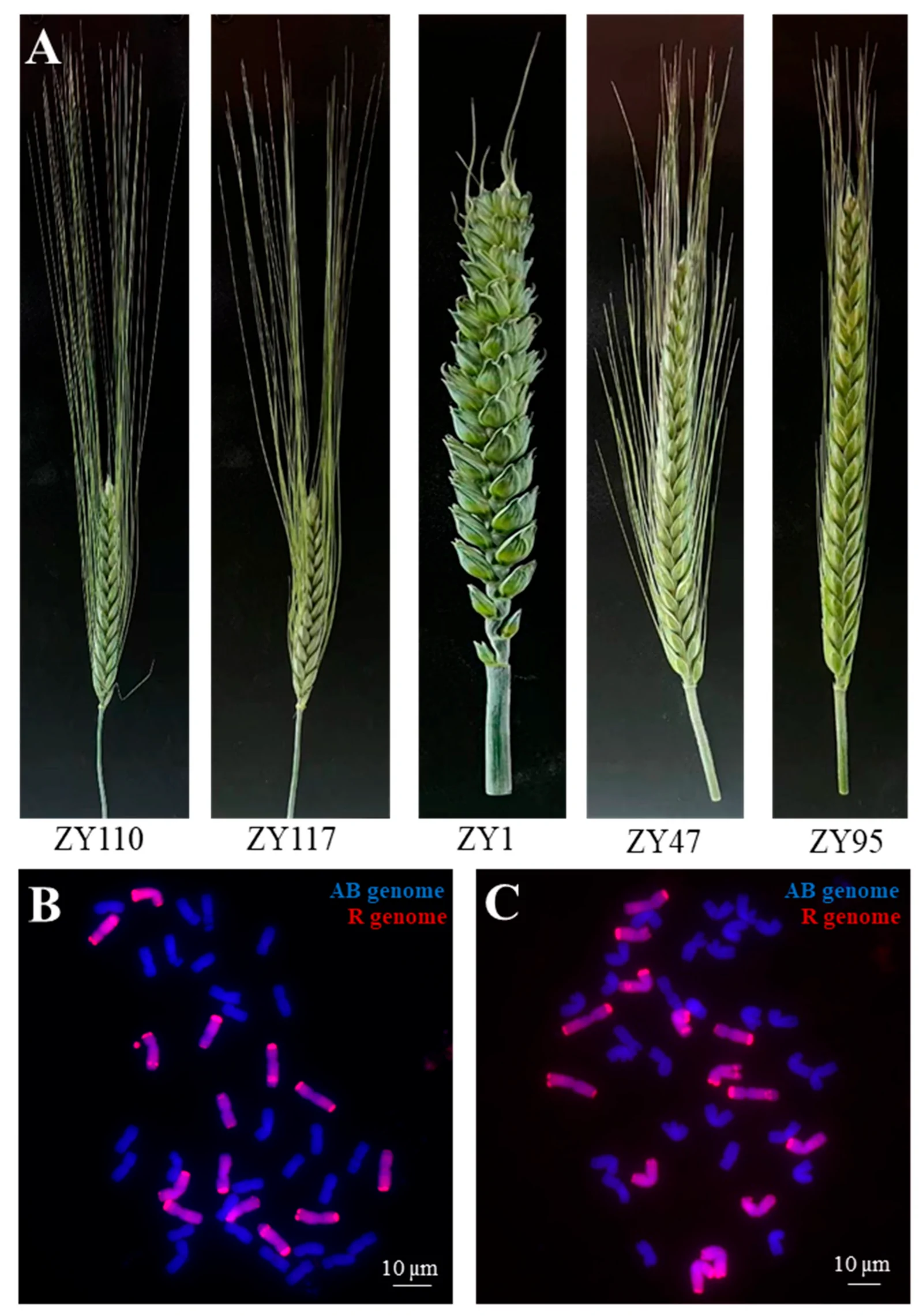
Morphological and cytological identification of wheat–rye amphiploids by genomic in situ hybridization (GISH). (**A**) Spike morphology of the amphiploids accessions (ZY47 and ZY95) compared with other resistant wheat accessions. GISH identification of (**B**) ZY47 and (**C**) ZY95 showing their distinct chromosomal constitutions. Rye genomic DNA was labeled with Atto-550 and produced a red GISH signal, whereas the blue fluorescence corresponds to DAPI counterstaining of the chromosomes. Red signals therefore identify rye-derived chromatin.

Based on plant and spike morphology, two of the five accessions, ZY47 and ZY95, were morphologically similar to triticale and exhibited a significantly later flowering time than other wheat accessions in the field (Figure 3A and Figure S1A). To confirm their genomic composition and elucidate the basis of these phenotypic differences, we performed genomic in situ hybridization (GISH) analysis. The results confirmed that both accessions, ZY47 and ZY95, possess a total of 42 chromosomes with the genome constitution AABBRR, containing a complete set of 14 rye chromosomes (Figure 3B,C and Figure S1B,C). For further characterization, we are currently crossing these two amphiploid lines with CM42*ph1b* and SM830*Ph1b* to generate rye translocation lines, which will facilitate the mapping and characterization of resistance gene(s) derived from the R genome.

### 3.4. Genetic Analysis and BSA of Resistance Genes

To further investigate the genetic basis of powdery mildew resistance in the five highly resistant wheat accessions, they were crossed with susceptible wheat cultivars Kronos, Langdon, CM42, SM830*Ph1b* and CM42*Ph1b* to develop F_1_, and F_2_ progeny. Seedlings of F_1_ and F_2_ populations were inoculated with powdery mildew. The F_1_ plants of two Central Asian accessions (ZY110 × Kronos and ZY117 × Langdon) showed partial resistance, whereas ZY1 × CM42 was susceptible, and two F_1_ plants of wheat–rye amphiploids (ZY47 × CM42*ph1b* and ZY95 × SM830*ph1b*) exhibited moderate resistance (Figure S2). ZY110 × Kronos and ZY117 × Langdon F_2_ populations were segregated in ratios consistent with a 3:1 resistant:susceptible ratio, indicating that a single dominant *Pm* resistance gene or genes confer resistance in each accession and that the ZY1 × CM42 F_2_ population segregated in a 1:3 resistant:susceptible ratio, indicating a single recessive *Pm* resistance gene or genes confer resistance in that accession (Table 1). BSA using 55K SNP-array genotyping revealed 530 and 664 high-quality SNPs between the R and S bulks, distributed across all 14 chromosomes (AA and BB genomes) in the ZY110 × Kronos and ZY117 × Langdon populations, respectively. A large proportion of these SNPs in both populations was detected on chromosome 2B, with enrichment in the terminal interval (6.88–35.26 Mb and 8.26 Mb–33.95 Mb) of arm 2BS (Figure S3A,B).

### 3.5. Molecular Marker Detection in Resistant Wheat Accessions and Linkage Analysis Through F_2_ Segregating Population

Upon investigating the relationship between resistance and specific *Pm* resistance gene(s), our results highlight the key findings: two durum wheat accessions (ZY110 and ZY117) contained *Pm68*, and ZY1 possessed a candidate *Pm26* allelic variant. To determine whether known rye-derived powdery mildew resistance genes are present in wheat–rye amphiploid lines, markers for all cloned *Pm* resistance genes, including the rye-derived gene markers *Pm8*, *Pm17*, and *PmTR1*/*PmTR3*, were tested, but none yielded the expected diagnostic amplicon in ZY47 or ZY95. These results suggest that ZY47 and ZY95 accessions are candidate sources of potentially novel rye-derived powdery mildew resistance (Table S5). Such candidate sources of potentially novel rye-derived powdery mildew resistance may represent valuable genetic resources for diversifying powdery mildew resistance in wheat breeding programs.

**Figure 4:**
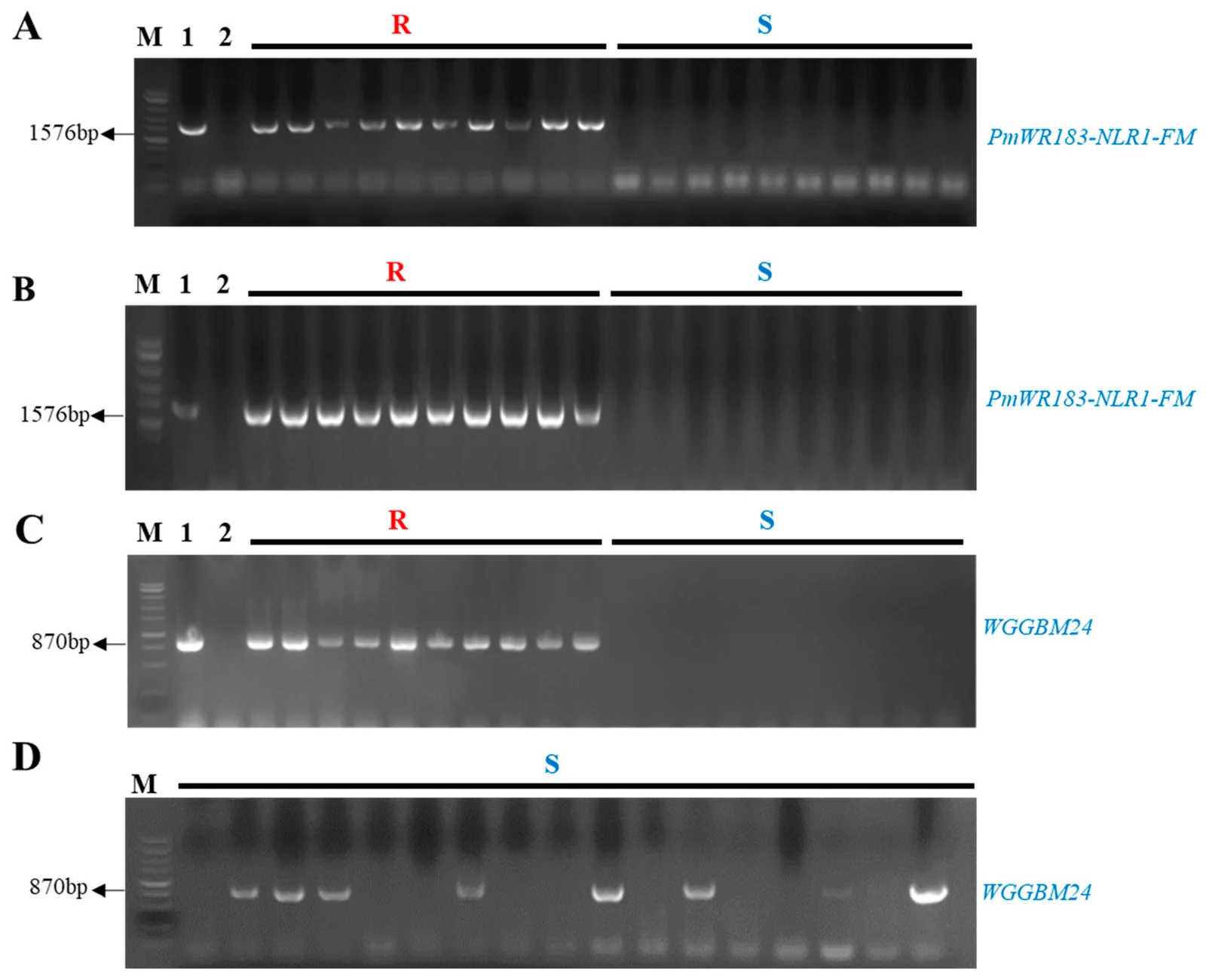
PCR amplification of gene-specific markers. (**A**) Lane 1, ZY117; lane 2, Xuezao; lanes 3–12, ZY117 F_2_ resistant plants; lanes 13–22, ZY117 F_2_ susceptible plants. (**B**) Lane 1, ZY110; lane 2, Xuezao; lanes 3–12, ZY110 F_2_ resistant plants; lanes 13–22, ZY110 F_2_ susceptible plants. (**C**) Lane 1, ZY1; lane 2, Xuezao; lanes 3–12, ZY1 F_2_ resistant plants; lanes 13–22, ZY1 F_2_ homozygous susceptible plants. (**D**) Lanes 23–39, ZY1 F_2_ susceptible homozygous (no band) and heterozygous (with band) plants.

To verify the *Pm* resistance gene(s), three molecular markers detected in the highly resistant wheat accessions were used to examine co-segregation of resistance in their corresponding F_2_ segregating populations. The *Pm68* allele STS-marker *PmWR183-NLR1-FM* was confirmed to be linked with powdery mildew resistance in ZY110 × Kronos and ZY117 × Langdon F_2_ populations, each consisting of 60 individual plants. All 43 resistant plants of ZY110 × Kronos and all 41 resistant plants of ZY117 × Langdon carried the marker band, while all 17 and 19 susceptible plants lacked it, respectively, consistent with dominant monogenic inheritance that confers resistance. Genotyping of 60 F_2_ individuals revealed zero recombinants between the marker and the resistance phenotype in both populations, corresponding to a genetic distance of 0 cM (95% CI: 0–5 cM) (Figure 4). Molecular marker analysis of the ZY1 × CM42 F_2_ population (N = 79) confirmed complete co-segregation between the *WGGBM24* marker for *Pm26* and the recessive monogenic inheritance model. All 15 resistant F_2_ plants carried the marker band, whereas among the 64 susceptible plants, the marker band was present in 42 heterozygous susceptible individuals and absent in 22 homozygous susceptible individuals, based on their F_2:3_ phenotypes, consistent with recessive monogenic inheritance. No phenotype–marker recombinants were observed among the 79 F_2_ individuals, corresponding to an observed recombination frequency of 0% and an observed genetic distance of 0 cM (95% CI: 0–3.8 cM). Together, these results support that these functional markers can be used for *Pm* resistance genes identification and marker-assisted selection in Central Asian wheat breeding programs.

### 3.6. Sequence Analysis of Known Pm Resistance Genes

To characterize the allelic variants of the *Pm* resistance genes in the highly resistant accessions, we cloned the homologous *Pm* sequences from two *Pm68*-carrying accessions and one accession carrying a candidate *Pm26* allelic variant. These sequences were then aligned with the reference sequences of *Pm68* (GenBank accession Nos. PQ655406.1 and PQ655412.1 for *Pm68NLR-1* and *Pm68NLR-2*, respectively) and *Pm26* (GenBank accession Nos. PP781951.1 and PP781952.1 for *Pm26-TdCNL1* and *Pm26-TdCNL5*, respectively). Sequence alignment revealed that the two durum wheat accessions, ZY110 and ZY117, which both harbor *Pm68*, share identical nucleotide sequences with the reference sequences. However, ZY1-*CNL1* was 99.63% identical to *Pm26-TdCNL1*, whereas ZY1-*CNL5* was only 92.30% identical to *Pm26-TdCNL5* (Supplementary Data S1).

### 3.7. Agronomic Trait Evaluations to Determine Breeding Potential of Resistance Donor Line

**Figure 5:**
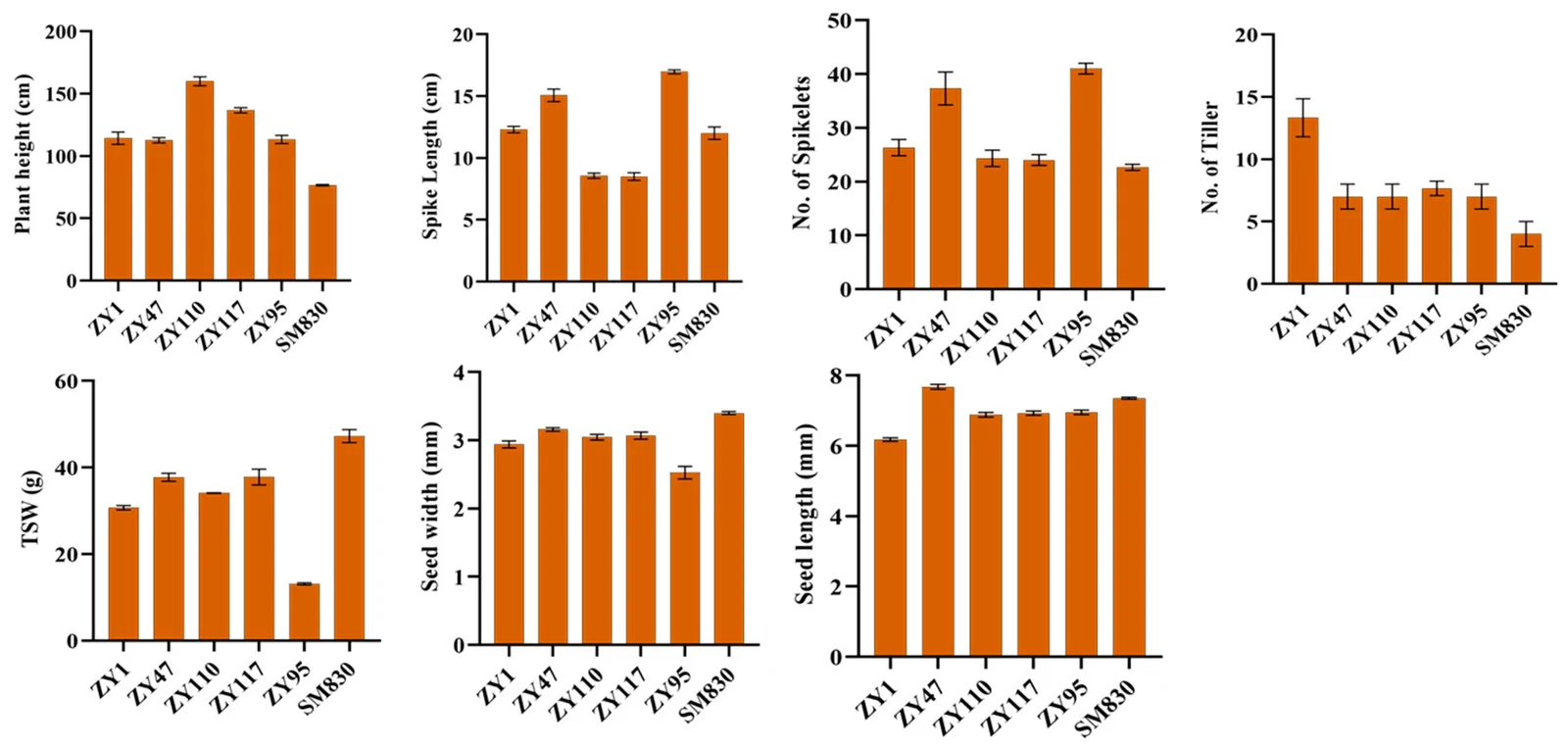
Comparisons of agronomic traits of five resistant accessions and SM830 (Sichuan wheat cultivar) as a control. Data presented as mean ± SD.

During the 2025–2026 wheat cropping season, the agronomic parameters of the five highly resistant wheat accessions were assessed at the maturity stage. The observed values of several agronomic and yield-related traits varied among the resistant accessions (Figure 5). ZY1 exhibited a higher tillering capacity, ZY95 produced the longest spikes and the greatest number of spikelets per spike, ZY47 and ZY117 showed a higher thousand-seed weight, and ZY47 also exhibited the longest seed length (Figure 5). Meanwhile, ZY110 displayed the greatest plant height, whereas ZY95 had the lowest thousand-seed weight (TSW). These preliminary data suggest that ZY47, ZY1, and ZY117, which combine broad-spectrum powdery mildew resistance with traits such as high tillering, a large seed size, and a high thousand-seed weight, may serve as promising donor parents for introducing resistance into elite wheat backgrounds. Although ZY95 exhibited desirable spike architecture, its lower thousand-seed weight suggests that additional breeding efforts, such as backcrossing with elite wheat cultivars, will be required to improve its agronomic performance.

## 4. Discussion

To speed up the breeding program of resistant germplasm, elucidating the constitution of resistance genes in these germplasms is critical. Although molecular marker detection is an efficient method for detecting the presence or absence of *Pm* resistance genes in wheat accessions, detecting only the resistant germplasms can produce false positives [5,54]. Furthermore, genetic analysis of segregating populations is essential for verifying marker linkage and accurately identifying the disease resistance genes present in resistant germplasm. BSA is also a quick method for the initial mapping of causal genes in the segregating populations. Sequencing can then detect allelic variations; following this process, we identified *Pm68* in two durum wheat accessions and a candidate *Pm26* allelic variant in one common wheat accession. Genetic analysis showed that resistance in the ZY110 and ZY117 populations is controlled by a single dominant gene, whereas resistance in ZY1 is controlled by a single recessive gene. Additionally, based on marker analysis, two accessions (AABBRR) are candidate sources of potentially novel rye-derived powdery mildew resistance.

Durum wheat possesses wide morphological and genetic diversity and is especially rich in disease resistance genes, making it an excellent resource for identifying novel resistance genes [55,56]. *Pm68* was mapped to the terminal segment of 2BS in the Greek durum line TRI 1796 and was found to be effective against all 22 *Bgt* isolates tested [57]; it is conferred by a pair of linked NLRs, *Pm68-1* and *Pm68-2*, with *Pm68-2* positively modulating the activation of *Pm68-1* [20]. TRI 1796 harbors a ~1.6 Mb interval of DNA that is quite unique from that of durum wheat (Svevo) but closely matches that of wild emmer (Zavitan), indicating that this particular region introgressed from a restricted wild emmer subgroup known as judaicum, the same group that includes Zavitan. Marker screening demonstrated that 6 of 120 durum accessions (5%) and 26 of 118 wild emmer (WEW) accessions (~22%) harbor the *Pm68* variant. Importantly, all six durum lines only harbored the functional allelic variant A that is similar to *Pm68*, whereas the WEW accessions exhibited far greater allelic diversity (Haplotypes A–I). Importantly, Haplotype F is similar to the independently reported resistance gene *MlIW39* [38], with only two amino acid changes, whereas the non-functional Zavitan haplotype (C) carries a premature stop codon in *Pm68-2*. The presence of *Pm68* in our Central Asian durum accessions indicates that it has spread well beyond its original Greek source and may have resulted from introgression, making it an important resistance resource for the region. Similarly, Wang et al. [37] detected *Pm68* in two *Triticum aestivum* subsp. *spelta* accessions, confirming that this gene is also present in hexaploid wheat relatives. Furthermore, it has been reported that *Pm68* introgression into Chinese elite wheat cultivar YM158 shows no deleterious linkage drag or adverse effects on key agronomic parameters, indicating its potential for disease resistance breeding in both durum and common wheat [20]. This is consistent with our observation that ZY110 and ZY117 maintained good agronomic performance while exhibiting strong powdery mildew resistance.

Zhu et al. [53] reported that *Pm26* (*MLIW170*/*Pm26*), originally identified in wild emmer wheat (*Triticum dicoccoides*, AABB) accession TTD140, encodes a pair of genetically linked NLRs, *TdCNL1* and *TdCNL5.* Using the diagnostic markers *WGGBM24* (*TdCNL1*) and *WGGBY25* (*TdCNL5*), they detected *Pm26* in only 39 accessions from a panel comprising 442 wild emmer accessions, 125 cultivated emmer accessions, 230 durum accessions, 262 Chinese wheat lines from a minicore collection, 636 additional Chinese wheat lines and 394 landraces from around the world, and all of the positive accessions were wild emmer. Detection of the candidate *Pm26* allelic variant in the common wheat accession ZY1 by *WGGBM24* suggests that this locus may also be present in hexaploid wheat, potentially extending its known distribution beyond tetraploid wheat. However, functional validation is required to confirm whether the candidate *Pm26* allelic variant represents a functional *Pm26* allele. ZY1-*CNL1* is homologous to *Pm26-TdCNL1* but exhibits sequence variations, and this result contradicts the previous claim that *TdCNL1* orthologs are absent in cultivated wheat genomes. Moreover, ZY1-*CNL5* was only 92.30% identical to *Pm26-TdCNL5*, which is consistent with the finding that *TdCNL5* is highly conserved across wheat and related species, with sequence identities ranging from 70.46% to 99.39%. When *Pm26* was first described in TTD140, the F_1_ plants were susceptible to all isolates tested and the segregation ratios indicated a single recessive allele [58], which is precisely the pattern seen in the ZY1 × CM42 population. The SNPs detected in ZY1 relative to the cloned *Pm26* sequence suggest that this accession may carry a candidate *Pm26* allelic variant. *Pm42*, also located on the terminal segment of 2BS, is likewise a single recessive gene [59], indicating that recessive resistance is not unusual at this region. Recently, Zhang et al. [60] reported the successful introgression of *Pm26* into common wheat introgression line 7D72, confirming that *Pm26* can be transferred from wild emmer to hexaploid wheat without observable linkage drag, while also improving agronomic traits such as grain length and thousand-kernel weight. However, whether the candidate *Pm26* allelic variant is functional remains unclear. Functional validation of the candidate *Pm26* allelic variant through the use of transgenes, CRISPR-Cas9, and EMS mutants, along with mapping and cloning, is needed to determine whether it functionally differs from the cloned allele.

Rye (*Secale cereale*, 2n = 2x = 14, RR), a tertiary gene pool species, shows high genetic heterogeneity and is a valuable genetic resource for disease resistance in wheat [61]. Triticale (×*Triticosecale* Wittmack), a synthetic amphiploid derived from wheat–rye hybridization, possesses numerous disease resistance genes and can be crossed with wheat more readily than rye, and thereby serves as a promising genetic resource for a wheat breeding program [62]. Among the rye-derived resistance genes, *PmTR1* and *PmTR3* originate from rye chromosome 6RS in wheat–rye lines TR1 and TR3, and confer broad-spectrum powdery mildew resistance similar to *Pm12* and *Pm21*, although with different modes of action: *PmTR1* provides age-related resistance from the three-leaf stage onward, while *PmTR3* provides all-stage resistance [26]. Similarly, the wheat-rye 2R (2D) substitution line YT9 provides powdery mildew resistance starting from the three-leaf stage, and the resistance gene *PmYT9* was delimited to a 14.55 Mb interval on 2RL [27]. The most notable finding of the present study is that ZY47 and ZY95 displayed all-stage resistance to *Bgt* isolate E09 and multiple isolates, yet none of the known *Pm* resistance gene*s* were detected. Markers for rye-derived *Pm8*, *Pm17*, and *PmTR1*/*PmTR3* also failed to amplify target bands. GISH confirmed their AABBRR genome constitution with 14 rye chromosomes, identifying them as wheat–rye amphiploids. The absence of markers for known rye-derived genes, together with their resistant phenotype, strongly suggests that ZY47 and ZY95 are candidate sources of potentially novel rye-derived powdery mildew resistance. As rye is a recognized reservoir of resistance genes, these candidate sources of potentially novel rye-derived powdery mildew resistance could help diversify powdery mildew resistance in wheat, especially as existing *Pm* resistance genes become ineffective. Further genetic mapping and functional characterization are required to determine whether the resistance in ZY47 and ZY95 is conferred by previously undescribed rye-derived *Pm* resistance gene(s).

In addition to the five highly resistant accessions, 48 wheat accessions displayed moderate resistance (IT-2). Although these accessions are not highly resistant, they may possess new resistance genes, minor QTLs, or recessive alleles that could be identified and deployed in breeding programs and could be used for gene stacking in wheat improvement to produce elite lines with all-stage resistance. Further investigation of these accessions using diagnostic markers and sequencing will enable the identification of candidate allelic variants that can be pyramided with known all-stage resistance genes to improve durability and to diversify the gene pool of wheat breeding programs in Central Asia. Field evaluations conducted in different years or across different locations are also needed to further validate the breeding value of these identified accessions. Functional validation of the candidate *Pm26* allelic variant will help us understand the molecular mechanisms and R gene evolution, and will facilitate the application of this allele in breeding. The *Pm68* allele was present in the durum accessions, whereas a candidate *Pm26* allelic variant was identified in the common wheat accession. These accessions exhibit favorable agronomic traits, are already used in the Central Asian wheat breeding system, and may offer greater breeding potential than wild emmer wheat alleles.

## 5. Conclusions

In summary, this study screened 192 wheat germplasm accessions and identified five resistant accessions. *Pm68* was identified in two durum accessions, ZY110 and ZY117, and a candidate *Pm26* allelic variant was found in common wheat ZY1. Most importantly, ZY47 and ZY95, which carry the rye R genome, showed no amplification of the tested markers for known *Pm* resistance genes, suggesting that these accessions are candidate sources of potentially novel rye-derived powdery mildew resistance. These findings offer valuable germplasm for breeding *Pm*-resistant wheat in Central Asia and enrich the resistance gene pool.

## Figures and Tables

**Table 1:** Segregation ratios in the F_2_ generations derived from crosses between resistant wheat accessions and a susceptible parent, following inoculation with *Blumeria graminis* f. sp. *tritici* (*Bgt*) isolate E09 at the seedling stage.

Parents	Generation	Ratio	Expected Ratio	X^2^	*p*	Inheritance
ZY1 × CM42	F_2_	R:S = 15:64	1:3	1.52	0.217	SR
ZY110 × Kronos	F_2_	R:S = 43:17	3:1	0.35	0.551	SD
ZY117 × Langdon	F_2_	R:S = 41:19	3:1	1.4223	0.233	SD

Note: R, resistant; S, susceptible; SR, single recessive (gene inheritance); SD, single dominant.

## Data Availability

The genotype data reported in this study are available in the Supplementary Data Sheet. The sequences of two *NLRs* (ZY1-*CNL1* and ZY1-*CNL5*) from wheat accession Jiyin Dong-1 were deposited in the GenBank database at NCBI under accession numbers PZ945933 for ZY1-*CNL1* and PZ945934 for ZY1-*CNL5*.

## Supplementary Materials

The following supporting information can be downloaded at: https://www.mdpi.com/article/10.3390/plants15203106/s1, Table S1. Material information and *Bgt* resistance evaluation of 192 Central Asian wheat accessions at seedling stage. Table S2. Virulence/avirulence profile and geographical origin of 13 *Blumeria graminis* f. sp. *tritici* (*Bgt*) isolates tested against 23 *Pm* genes or haplotypes. Table S3. Marker information of cloned *Pm* genes for marker detection in five resistant accessions. Table S4. Primer information used for cloning known *Pm* genes which are present in wheat accessions. All primers were designed based on the target gene-specific sequences. Table S5. Detection of Powdery mildew (*Pm*) resistance genes with molecular markers. +, gene present; -, gene absent. Figure S1. (A) Plant morphology of amphiploids, and Israeli common wheat cultivar (Ruta) (B) Karyotyping of ZY95 and (C) ZY47 revealed 42 chromosomes. Figure S2. Powdery mildew resistance evaluation of five F_1_ resistant Central Asian wheat accessions. 1: ZY1 × CM42, 2: ZY110 × Kronos, 3: ZY117 × LND, 4: ZY95 × SM830*ph1b*, 5: ZY47 × CM42*ph1b*. Figure S3. Distribution of polymorphic SNPs between resistant (R) and susceptible (S) pools on 14 wheat chromosomes based on SNP density plot. (A) ZY117 × Langdon (B) ZY110 × Kronos. Refs. [63,64,65,66,67,68,69,70,71,72,73,74,75,76,77,78,79,80,81,82,83,84,85,86] were cited in the Supplementary Materials.
