# Supplementary material for: Screening Central Asian Wheat Germplasm for Powdery Mildew Resistance and Molecular Characterization of Disease Resistance Genes
Source: Plants (Basel). 2026 Oct 10;15(20):3106. doi: 10.3390/plants15203106 (PMC13708471; doi:10.3390/plants15203106)
Supplement: Supplementary file 1 [file plants-15-03106-s001.zip › plants-4534955-supplementary/supplementary tables and figures.pdf]

Table S1. Material information and *Bgt* resistance evaluation of 192 Central Asian wheat accessions at seedling stage.

ZY Number	Accession Name	Country	IT Level (seedling stage)
ZY/1	Jiyin Dong-1	Kyrgyzstan	0
ZY/2	Jiyin Dong-4	Kyrgyzstan	4
ZY/3	Jiyin Dong-6	Kyrgyzstan	2
ZY/4	Jiyin Dong-9	Kyrgyzstan	4
ZY/5	Jiyin Dong-11	Kyrgyzstan	4
ZY/6	Jiyin Dong-14	Kyrgyzstan	4
ZY/7	Jiyin Dong-18	Kyrgyzstan	4
ZY/8	Jiyin Dong-24	Kyrgyzstan	4
ZY/9	Jiyin Dong-25	Kyrgyzstan	3
ZY/10	Jiyin Dong-29	Kyrgyzstan	4
ZY/11	Jiyin Dong-31	Kyrgyzstan	4
ZY/12	Jiyin Dong-37	Kyrgyzstan	4
ZY/13	Jiyin Dong-43	Kyrgyzstan	2
ZY/14	Jiyin Dong-47	Kyrgyzstan	2
ZY/15	Jiyin Dong-52	Kyrgyzstan	4
ZY/16	Jiyin Dong-55	Kyrgyzstan	4
ZY/17	Jiyin Dong-58	Kyrgyzstan	3
ZY/18	Jiyin Dong-84	Kyrgyzstan	3
ZY/19	Jiyin Dong-98	Kyrgyzstan	4
ZY/20	Jiyin Dong-104	Kyrgyzstan	4
ZY/21	Jiyin Dong-128	Kyrgyzstan	4
ZY/22	Jiyin Dong-132	Kyrgyzstan	4
ZY/23	Jiyin Dong-133	Kyrgyzstan	4
ZY/24	Jiyin Dong-139	Kyrgyzstan	3
ZY/25	Jiyin Dong-140	Kyrgyzstan	4
ZY/26	Jiyin Dong-145	Kyrgyzstan	4
ZY/27	Jiyin Dong-150	Kyrgyzstan	4
ZY/28	Jiyin Dong-158	Kyrgyzstan	4
ZY/29	Jiyin Dong-164	Kyrgyzstan	3
ZY/30	Jiyin Dong-174	Kyrgyzstan	4
ZY/31	Jiyin Dong-176	Kyrgyzstan	4
ZY/32	Jiyin Dong-177	Kyrgyzstan	4
ZY/33	Jiyin Dong-181	Kyrgyzstan	4
ZY/34	Jiyin Dong-218	Kyrgyzstan	4
ZY/35	Jiyin Dong-222	Kyrgyzstan	4
ZY/36	Jiyin Dong-223	Kyrgyzstan	4
ZY/37	Jiyin Dong-225	Kyrgyzstan	2
ZY/38	Jiyin Dong-228	Kyrgyzstan	4
ZY/39	Jiyin Dong-233	Kyrgyzstan	4
ZY/40	Jiyin Dong-240	Kyrgyzstan	3
ZY/41	Jiyin Dong-241	Kyrgyzstan	2
ZY/42	Jiyin Dong-244	Kyrgyzstan	4

ZY/43	Jiyin Dong-246	Kyrgyzstan	4
ZY/44	Jiyin Dong-247	Kyrgyzstan	4
ZY/45	Jiyin Dong-251	Kyrgyzstan	4
ZY/46	Jiyin Dong-252	Kyrgyzstan	4
ZY/47	Jiyin Dong-255	Kyrgyzstan	0
ZY/48	Jiyin Dong-256	Kyrgyzstan	4
ZY/49	Jiyin Dong-258	Kyrgyzstan	4
ZY/50	Jiyin Dong-264	Kyrgyzstan	4
ZY/51	Jiyin Dong-269	Kyrgyzstan	4
ZY/52	Jiyin Dong-270	Kyrgyzstan	4
ZY/53	Jiyin Dong-273	Kyrgyzstan	3
ZY/54	Hayin-2013-XM-1	Kazakhstan	3
ZY/55	Hayin-2013-XM-2	Kazakhstan	4
ZY/56	Hayin-2013-XM-3	Kazakhstan	4
ZY/57	Hayin-2013-XM-4	Kazakhstan	3
ZY/58	Hayin-2013-XM-5	Kazakhstan	4
ZY/59	Tayin XM2012-3	Tajikistan	3
ZY/60	Tayin XM2012-5	Tajikistan	4
ZY/61	Tayin XM2012-23	Tajikistan	4
ZY/62	Tayin XM2012-23	Tajikistan	4
ZY/63	Tayin XM2012-41	Tajikistan	3
ZY/64	Tayin XM2012-42	Tajikistan	4
ZY/65	Tayin XM2012-44	Tajikistan	2
ZY/66	Tayin XM2012-47	Tajikistan	3
ZY/67	Tayin XM2012-59	Tajikistan	3
ZY/68	Tayin XM2012-65	Tajikistan	4
ZY/69	Tayin XM2012-66	Tajikistan	4
ZY/70	Jiyin C-XM2012-9	Kyrgyzstan	2
ZY/71	Jiyin C-XM2012-10	Kyrgyzstan	2
ZY/72	Jiyin C-XM2012-11	Kyrgyzstan	2
ZY/73	Jiyin C-XM2012-13	Kyrgyzstan	2
ZY/74	Jiyin C-XM2012-14	Kyrgyzstan	2
ZY/75	Jiyin C-XM2012-15	Kyrgyzstan	2
ZY/76	Jiyin C-XM2012-16	Kyrgyzstan	2
ZY/77	Jiyin C-XM2012-17	Kyrgyzstan	4
ZY/78	Jiyin C-XM2012-18	Kyrgyzstan	4
ZY/79	Jiyin C-XM2012-19	Kyrgyzstan	3
ZY/80	Jiyin C-XM2012-20	Kyrgyzstan	2
ZY/81	Jiyin C-XM2012-21	Kyrgyzstan	3
ZY/82	Jiyin C-XM2012-22	Kyrgyzstan	3
ZY/83	Jiyin C-XM2012-23	Kyrgyzstan	2
ZY/84	Jiyin C-XM2012-34	Kyrgyzstan	4
ZY/85	Jiyin C-XM2012-36	Kyrgyzstan	2
ZY/86	Jiyin C-XM2012-37	Kyrgyzstan	3
ZY/87	Jiyin C-XM2012-38	Kyrgyzstan	2
ZY/88	Jiyin C-XM2012-42	Kyrgyzstan	4
ZY/89	Jiyin C-XM2012-42	Kyrgyzstan	2

ZY/90	Jiyin C-XM2012-44	Kyrgyzstan	2
ZY/91	Jiyin C-XM2012-46	Kyrgyzstan	4
ZY/92	Jiyin C-XM2012-47	Kyrgyzstan	4
ZY/93	Jiyin C-XM2012-48	Kyrgyzstan	3
ZY/94	Jiyin C-XM2012-50	Kyrgyzstan	2
ZY/95	Jiyin C-XM2012-51	Kyrgyzstan	0
ZY/96	Jiyin C-XM2012-52	Kyrgyzstan	2
ZY/97	Jiyin C-XM2012-54	Kyrgyzstan	3
ZY/98	Jiyin C-XM2012-60	Kyrgyzstan	4
ZY/99	Tayin XM2012-1	Tajikistan	3
ZY/100	Tayin XM2012-2	Tajikistan	2
ZY/101	Tayin XM2012-2	Tajikistan	3
ZY/102	Tayin XM2012-3	Tajikistan	2
ZY/103	Tayin XM2012-4	Tajikistan	4
ZY/104	Tayin XM2012-5	Tajikistan	4
ZY/105	Tayin XM2012-6	Tajikistan	4
ZY/106	Tayin XM2012-7	Tajikistan	2
ZY/107	Tayin XM2012-8	Tajikistan	3
ZY/108	Tayin XM2012-8	Tajikistan	4
ZY/109	Tayin XM2012-10	Tajikistan	2
ZY/110	Tayin XM2012-11	Tajikistan	0
ZY/111	Tayin XM2012-13	Tajikistan	4
ZY/112	Tayin XM2012-13	Tajikistan	3
ZY/113	Tayin XM2012-14	Tajikistan	2
ZY/114	Tayin XM2012-15	Tajikistan	4
ZY/115	Tayin XM2012-15	Tajikistan	4
ZY/116	Tayin XM2012-16	Tajikistan	4
ZY/117	Tayin XM2012-17	Tajikistan	0
ZY/118	Tayin XM2012-18	Tajikistan	2
ZY/119	Tayin XM2012-19	Tajikistan	3
ZY/120	Tayin XM2012-20	Tajikistan	3
ZY/121	Tayin XM2012-21	Tajikistan	3
ZY/122	Tayin XM2012-22	Tajikistan	3
ZY/123	Tayin XM2012-24	Tajikistan	2
ZY/124	Tayin XM2012-25	Tajikistan	4
ZY/125	Tayin XM2012-26	Tajikistan	4
ZY/126	Tayin XM2012-29	Tajikistan	3
ZY/127	Tayin XM2012-31	Tajikistan	3
ZY/128	Tayin XM2012-32	Tajikistan	3
ZY/129	Tayin XM2012-33	Tajikistan	3
ZY/130	Tayin XM2012-34	Tajikistan	3
ZY/131	Tayin XM2012-37	Tajikistan	4
ZY/132	Tayin XM2012-38	Tajikistan	3
ZY/133	Tayin XM2012-39	Tajikistan	2
ZY/134	Tayin XM2012-40	Tajikistan	2
ZY/135	Tayin XM2012-43	Tajikistan	4
ZY/136	Tayin XM2012-45	Tajikistan	4

ZY/137	Tayin XM2012-47	Tajikistan	3
ZY/138	Tayin XM2012-48	Tajikistan	4
ZY/139	Tayin XM2012-48	Tajikistan	4
ZY/140	Tayin XM2012-50	Tajikistan	2
ZY/141	Tayin XM2012-51	Tajikistan	2
ZY/142	Tayin XM2012-52	Tajikistan	2
ZY/143	Tayin XM2012-53	Tajikistan	2
ZY/144	Tayin XM2012-54	Tajikistan	3
ZY/145	Tayin XM2012-55	Tajikistan	2
ZY/146	Tayin XM2012-56	Tajikistan	2
ZY/147	Tayin XM2012-56	Tajikistan	2
ZY/148	Tayin XM2012-57	Tajikistan	4
ZY/149	Tayin XM2012-58	Tajikistan	4
ZY/150	Tayin XM2012-59	Tajikistan	4
ZY/151	Tayin XM2012-60	Tajikistan	4
ZY/152	Tayin XM2012-61	Tajikistan	4
ZY/153	Tayin XM2012-62	Tajikistan	4
ZY/154	Tayin XM2012-63	Tajikistan	4
ZY/155	Tayin XM2012-64	Tajikistan	3
ZY/156	Tayin XM2012-65	Tajikistan	4
ZY/157	Tayin XM2012-66	Tajikistan	4
ZY/158	Tayin XM2012-67	Tajikistan	2
ZY/159	Tayin XM2011-1	Tajikistan	4
ZY/160	Tayin XM2011-2	Tajikistan	4
ZY/161	Hayin XM2012-1	Kazakhstan	4
ZY/162	Hayin XM2012-2	Kazakhstan	4
ZY/163	Hayin XM2012-3	Kazakhstan	2
ZY/164	Hayin XM2012-4	Kazakhstan	3
ZY/165	Hayin XM2012-5	Kazakhstan	3
ZY/166	Hayin XM2012-6	Kazakhstan	3
ZY/167	Hayin XM2012-7	Kazakhstan	4
ZY/168	Hayin CXMClM-1	Kazakhstan	4
ZY/169	Hayin CXMClM-2	Kazakhstan	4
ZY/170	Hayin CXMClM-3	Kazakhstan	4
ZY/171	Hayin CXMClM-3	Kazakhstan	4
ZY/172	Hayin CXMClM-4	Kazakhstan	3
ZY/173	Hayin CXMClM-5	Kazakhstan	4
ZY/174	Hayin CXMClM-6	Kazakhstan	2
ZY/175	Hayin CXMClM-7	Kazakhstan	2
ZY/176	Hayin CXMClM-8	Kazakhstan	2
ZY/177	Hayin CXMClM-9	Kazakhstan	4
ZY/178	Hayin CXMClM-10	Kazakhstan	4
ZY/179	Hayin CXMClM-11	Kazakhstan	2
ZY/180	Hayin CXMClM-12	Kazakhstan	2
ZY/181	Hayin CXMClM-13	Kazakhstan	4
ZY/182	Hayin CXMClM-14	Kazakhstan	2
ZY/183	Hayin ASTNCXM2012-1	Kazakhstan	3

ZY/184	Hayin ASTNCXM2012-2	Kazakhstan	2
ZY/185	Hayin ASTNCXM2012-3	Kazakhstan	3
ZY/186	Hayin ASTNCXM2012-4	Kazakhstan	3
ZY/187	Hayin ASTNCXM2012-5	Kazakhstan	3
ZY/188	Hayin ASTNCXM2012-6	Kazakhstan	4
ZY/189	Hayin ASTNCXM2012-7	Kazakhstan	2
ZY/190	Hayin ASTNCXM2012-8	Kazakhstan	2
ZY/191	Hayin ASTNCXM2012-9	Kazakhstan	4
ZY/192	Hayin ASTNCXM2012-10	Kazakhstan	4

Note: Infection types (ITs) were scored on a 0–4 scale: 0, highly resistant (no visible infection symptoms); 1, resistant (necrotic flecks with no visible sporulation); 2, moderately resistant (small colonies with absent or sparse sporulation, diameter ≤ 1 mm); 3, moderately susceptible (moderate mycelial growth and sporulation, diameter ≥ 1 mm); 4, highly susceptible (well-developed mycelium and abundant sporulation diameter ≥ 1 mm).

Table S2. Virulence/avirulence profile and geographical origin of 13 *Blumeria graminis* f. sp. *tritici* (*Bgt*) isolates tested against 23 *Pm* genes or haplotypes.

**Isolates name**	**E09**	**E26**	**WJ**	**SZS**	**GH**	**DY**	**SN**	**SD-JN**	**DY-ZJ**	**NC**	**JF**	**AS**	**BMM1**
Geographical origin/ *Pm* genes or Haplotypes	Beijing, China	Unknown	Wenjiang, Chengdu, Sichuan, China	Jinjiang, Chengdu, Sichuan, China	Guanghan, Deyang, Sichuan, China	Dayi, Chengdu, Sichuan, China	Shehong, Suining, Sichuan, China	Changqing, Jinan, Shandong, China	Zhongjiang, Deyang, Sichuan, China	Langzhong, Nanchong, Sichuan, China	Jifeng, Deyang, Sichuan, China	Xixiu, Anshun, Guizhou, China	Jinjiang, Chengdu, Sichuan, China
Pm3f	2	3	3	0	2	3	3	2	0	1	2	2	0
Pm3g	0	3	1	0	0	3	2	2	0	2	2	1	3
Pm5e	1	3	0	3	0	0	0	0	0	1	2	3	0
Pm6	4	1	0	0	0	1	1	0	0	2	1	1	0
Pm13	0	1	0	0	0	1	0	0	0	0	0	1	1
Pm16	0	2	0	3	0	1	0	0	0	2	1	2	1
Pm17	0	2	1	3	0	1	0	1	0	0	2	0	0
Pm19	3	3	1	3	2	0	1	1	2	1	2	3	0
Pm21	0	0	0	0	0	0	0	0	0	0	0	1	0
Pm24	2	1	1	0	0	2	1	0	0	1	2	1	2
Pm25	1	0	1	0	0	0	0	0	3	0	3	3	0
Pm26	1	1	2	1	1	2	0	0	0	2	2	0	2
Pm32	3	3	3	0	3	2	0	2	2	0	3	3	2
Pm36	2	2	3	2	1	1	0	1	1	1	2	1	1
Pm41	0	1	0	0	0	0	0	0	1	0	0	0	0
Pm48	2	2	3	3	3	3	1	2	0	2	2	1	0
Pm56	0	1	0	0	1	0	0	0	0	0	0	0	0
Pm57	1	0	0	0	0	1	0	0	0	0	0	0	0
Pm60	2	1	2	0	3	0	0	0	0	0	0	1	0
Pm60b	4	0	3	2	1	2	2	0	1	2	2	4	0
Pm64	2	2	2	3	2	2	0	0	2	3	3	3	2

Isolates name	E09	E26	WJ	SZS	GH	DY	SN	SD-JN	DY-ZJ	NC	JF	AS	BMM1
Pm68	0	2	2	1	1	2	2	0	2	2	2	1	2
Pm69	0	2	0	0	1	0	0	1	1	1	0	0	1

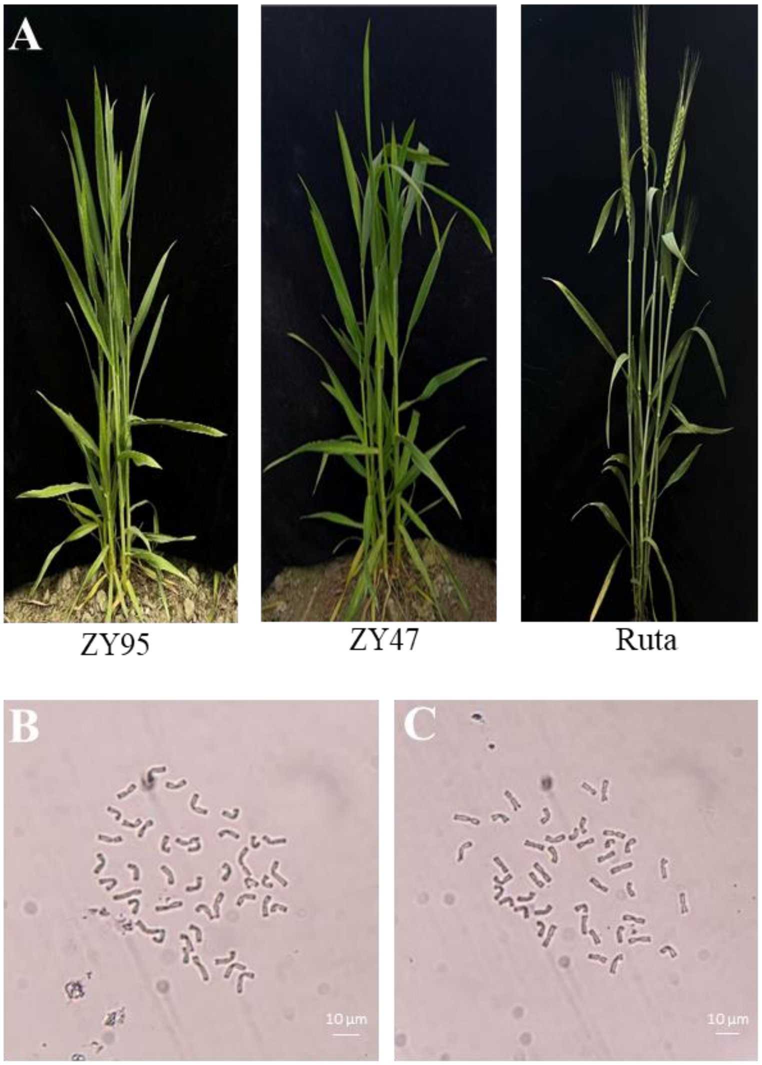

Figure S1. (A) Plant morphology of amphiploids, and Israeli common wheat cultivar (Ruta) (B) Karyotyping of ZY95 and (C) ZY47 revealed 42 chromosomes.

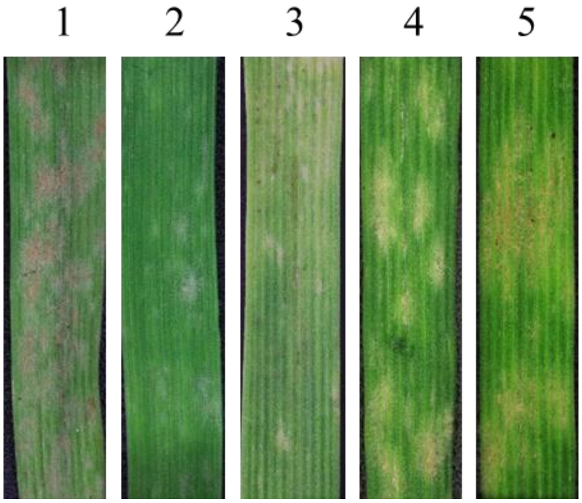

Figure S2. Powdery mildew resistance evaluation of five F_1 resistant Central Asian wheat accessions at seedling stage. 1: ZY1 x CM42, 2: ZY110 x Kronos, 3: ZY117 x LND, 4: ZY95 x SM830*ph1b* , 5: ZY47 x CM42*ph1b*.

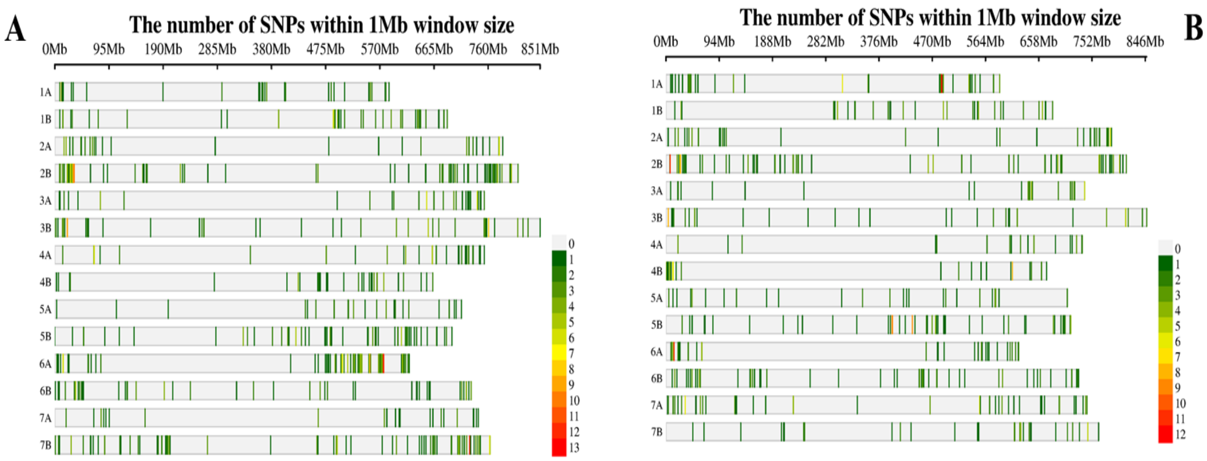

Figure S3. Distribution of polymorphic SNPs between resistant (R) and susceptible (S) pools on 14 wheat chromosomes based on SNP density plot. (A) ZY117 x Langdon (B) ZY110 x Kronos.

Table S3. Marker information of cloned *Pm* genes for marker detection in five resistant accessions.

Gene	Marker name	Forward primer (5′-3′)	Reverse primer (5′-3′)	Product length (bp)	Tm (°C)	References
Pm1a	*Pm1aSTS1*	CAATATAAACTTCAGATGTTCTAT TCTCAAAC	CTACATTGGCTATGCGTGTAGTC	333	53	Hewitt et al. [1]
Pm2	*Pm2b-map-3*	TGGTAACGAAGGTTGTCGCC	GCTCAATCTGAGAACCTT	150	58	Jin et al. [2]
Pm3b	*Pm3b/F*	GGCACAGACAAAGCTCTG	TCGAGTAGCTCGGGAATC	1382	54	Tommasini et al. [3]
Pm8	*Sfr43*	TGGCTTCCAACAGCCCTAGC	AGGCTTTTGCACCTTCTCTC	662	57	Hurni et al. [4]
Pm17	*DST156/DST157*	CCATATGGATTGATGAATGC	TCGGTTTAAATGCTGTTAC	699	51	Singh et al. [5]
Pm12	*6SS-04*	AGCTGTTTCCTTTCCAATGAGTAA	GCTGTGAATCCATTATGCTGTTT CA	722	54	Zhu et al. [6]
Pm21	*MBH1*	GCCATTATAGTCAAGAGTGCACT	AGCTCCTCTCGTTCTCC	330	53	He et al. [7]; Xing et al. [8]
Pm41	*WGGB427*	CAGATCTGAACCTACTCTGCTCT	GAGGGAGTTCATCACCCCAGTG GCAAAGCT	214/189	63-57	Li et al. [9]
Pm55a	*Pm55 qPCR*	CGGGAAAAGGAGAAACAAGAT	TCACACGGGTAGCGGAATGT	195	-	Lu et al. [10]
Pm60/Pm60a/Pm 60b MlIW172/MlWE 18/TdPm60	*M-Pm60-S1*	CTCACAGTTCCACACTGATAT	CTCCATCAATCTCAAGTTCTTCG	831	53	Wu et al. [11]; Wu et al. [12]; Li et al. [13]
Pm69	*uhw403*	GCTATCGCCATCTACCTATC	ATAAACTCCCGAGATGCG	549	53	Li et al. [14]
PmTR1/ PmTR3	*TR-D*	GATGTCTCCTCCTGCCTCAG	TTCGGTACAGGCCAGATTGA	226/244	53	Han et al. [15]
PmAeu1	*XAeuNLR1*	GATGCCTCTCAGGCCTCCGCTT	GGCATGACTCTTCTCTGGTGCCT	-	58	He et al. [16]
Pm6Sl	*pm6sl-1*	GCCGAGCTAACAACCTTCCTC	GCATGGGAATTAATGTTGTG	904	54	Ma et al. [17]
Pm5e	*WGGB202*	CTTCAATCGCGACAGGTCTG	TCGGTTTTGGTGGACTGGTA	257	56	Xie et al., [18]
Pm26/MlIW170	*WGGBM24*	CACATCTGGAAGGGCTAAACG	GTGCTGTTGAACTCTTCCAGG	870	54	Zhu et al. [19]
Pm4b	*JS717/JS718*	AGGTGGACATCCTAGGCGCT	GATCTGGGTACCACAGCACCG	926	58	Sánchez-Martín et al. [20]
Pm13	*AelMLKL-1*	TTCCTCCCGTCACCGATA	TGGCACTCCTGCTTGTTCT	399	53	Li et al. [21]; He et al. [22]
Pm24	*STS-Pm24*	TATGGTGTCATTTAAGGCTGAG	TTTCTCACATCCTCATCAAACC	171	53	Lu et al. [23]
WTK4	*JS693/JS671*	CTTTCATCCTCTGTCTCCCCC	TGGTGGTTGCATCATCCATAAGT	Full-length	-	Gaurav et al. [24]
Pm36	*WTK7-TM-FM*	CGGCTCCATTTCAAAGAAGA	CGGAGAGTTTTGGGATTGTG	1198	54	Li et al. [25]
Pm57	*STS-Pm57*	AGGGAGATATGCTTTGGTTACAT	CTCGTTGCTGAGAACACCACTT	530	55	Zhao et al. [26]
Pm38 (Yr18/Lr34/Sr57)	*cssfr1*	TTGATGAAACCAGTTTTTTTTCTA	GCCATTTAACATAATCATGATGG A	517	51	Lagudah et al. [27]
Pm46 (Yr46/Lr67/Sr55)	*SNP1-TM4*	F1: GAAGGTCGGAGTCAACGGATTTC ATCATCGGCAGGATCCTGCTTG	AACGTACGTAATCTTGCTTACTG A	-	-	Moore et al. [28]

Pm68	*Xdw08.9*	CTAGATGTCAGTCCAGATGTTG	GTTATGCTGCAGTAACTAACTCT C	156	53	He et al. [29]
MlIW39	*7IND2*	TCCCCATAATTTTGTCCTAACC	GGGGATCAGCCAACAGTTAG	218	54	Yang et al. [30]
PmWR183	*PmWR183-NLR1-FM*	TTCCCGTGAACGATGGAGATAC	CATTTCAACCCTCTCGCATT	1576	55	Zhang et al. [31]
	PmWR183-NLR2-FM	AAGGCTCTGCTGAGAGGATGA	TAGACTGCGGAAGCTTGTGA	863	54	
Pm6/Pm52	*Pm52-FM*	CCTGCACGCCCCTCTATTAC	TGGAACCTGGAAATGGCTCT	-	56	Wang et al. [32]
Pm37	*Pm37-FM*	TGTCAAACTTGCGGCTAT	TTTCCTGGTTTCGTAGCA	473	53	Jin et al. [33]
PmNCA6	*FM-NLR-*	TACACGCCAGACTCCCTAAC	GGACAACACCCTGCCATTG	276	54	Wan et al. [34]
PmD479	*PmD479-FM*	GTTCCATCTAGGTGACCGTACCT	TCATTCATTGTCACTGACAAGTA	285	56	Tang et al. [35]

Table S4. Primer information used for cloning known Pm genes which are present in wheat accessions.

Marker name	Forward Primer	Reverse Primer
Pm26-R1-1	CCGCTCCAAACAGATCTTGC	TGAACTGACGTGCAATAGAACT
Pm26-R1-2	ACAGTGCGTCTTCTGAGTTCA	CCACTAGATCCGGCACTGTT
Pm26-R1-3	AACAGTGCCGGATCTAGTGG	ACTACTCACTTTATCCCGACAGG
Pm26-R2	ATGGAGCTGGTGGTGGGA	CCATAGGTTCTCACTTTGGATC
Pm68-R1JCFL	ACGACTCAATAGGAGCGTTGGATAAATATCG	CCAGATACAGGAGGGGCTTG
Pm68-R2JCFL	ACACCGTGAATCCGCTGAAT	TGCGACAGACCTTGCAAGAT

Table S5. Detection of Powdery mildew (*Pm*) resistance genes with molecular markers. +, gene present; -, gene absent.

Pm genes	ZY1	ZY110	ZY117	ZY47	ZY95
Pm1a	-	-	-	-	-
Pm2	-	-	-	-	-
Pm3b	-	-	-	-	-
Pm8	-	-	-	-	-
Pm17	-	-	-	-	-
Pm12	-	-	-	-	-
Pm21	-	-	-	-	-
Pm41	-	-	-	-	-
Pm55a	-	-	-	-	-
Pm55b	-	-	-	-	-
Pm60a	-	-	-	-	-
Pm60	-	-	-	-	-
Pm60b	-	-	-	-	-
MlIW172/MlWE18/TdPm60	-	-	-	-	-
Pm69	-	-	-	-	-
PmTR1	-	-	-	-	-
PmTR3	-	-	-	-	-
PmAeu1	-	-	-	-	-
Pm6Sl	-	-	-	-	-
Pm5e	-	-	-	-	-
Pm26/MlIW170	+	-	-	-	-
Pm4b	-	-	-	-	-
Pm13	-	-	-	-	-
Pm24	-	-	-	-	-
WTK4	-	-	-	-	-
Pm36	-	-	-	-	-
Pm57	-	-	-	-	-
Pm38 (Yr18/Lr34/Sr57)	-	-	-	-	-
Pm46 (Yr46/Lr67/Sr55)	-	-	-	-	-
Pm68/MlIW39/PmW183	-	+	+	-	-
Pm6	-	-	-	-	-
Pm37/PmNCA6	-	-	-	-	-
PmD479	-	-	-	-	-

(A)

>ZY1-*CNL1*

ATGGCCCAGGCCATCGTCCACAGGTCACTTACAAAGCTCAGATCCGCCATTGGAGATGAGAAGAACACGATGTGCTCCAAGTTCGACGGTGA
CCTACGGGAAATGGTGGGGGTGCTGGAGGACATAGAGCCGGTGTTGGAGGACGTCGAGATACAGTCGTTGTGGAACAAGGACTCGCAGGAG
CGGGTGTGGTTGAGAAGGGTGAGGAACGCCTCCTACGGCATCTCCGACATGGTCGACGAGTTGAAGGACACGAGGCCTCCTGGCAAGGCAT
GTACTCGTACCTCTATCGTTCATCATACCTTCAATTTTACTTGCTCGATTGACTTCACGTTGATTAATCCCGCAGATGGCATGTATTTCCATGGT
ACTCAAAAGGAAGATCATGTCCAACAAGATGGAGGAGATGAAGGGAAAGCTTAGGATGTTACTACAGCAGGAGTGGCATCGGGCCTCTGTG
CCAGAATCTACTGGCGTCCAAGTCCAACCACCGTCTCCACCACCACATCCACTTTCAGAAACTGCTGGCGTTCAACCACTGTATCATCCACTG
CAACCTCCATCTTCACTTTCCACACCATGGTATCCACCTCCACCTCCAGTTTACATGTATCCACTTCCACTTTTCATACCACGGTATCCACCTCC
ACCTCCACCTCCACCTCCACCTCCACTTTACACAACACCTCCATTTTCACCAGTATTCAGTCGAGCTCTACCACTAAATAGAATAGTTTGGGGA
AGTGACGCGCATAAGTGGGTTGATAAAGAGAACATTGTAGGGAGGGGTGAAAATAAGCAGAGGATAATTGCTACACTATCTGCCACCGACA
TCATAGAAGGCCCTGTCATTCTTCCCATATTTGGTCTCGCCACAATAGGGAAGACAACCTTGGCAGCAATGATTTTCCAAGATACTGGCTTCG
GAGAATACAAGCTACGGATATGGGTGGATGTCACTCCAAAATTTGACTTGCCTAAAATAGGACATTACATAATCTCTAAAATAAAAGAGATC
AACTTAGAAACGGAACACCAGAGAGGCTACGGTAATCAAGTTTCGGAACAAGAGGCCAGCAAAGGTTCAGATGGGGCTTCTCAAATATCAG
AAGATGAGACCAGCCATGGTTCAGATGGGGCTTCTCAAATATCAGAAGATGAGACCAGCGAAGGTTTAGATGGGGCTTCTCAAGTATCAGAA
GAAGAGATCAACAAAGGTTCAGATGGGTTTTCTCAAGTACTAGCAGAAGAGATCAACAAAGGTACATGTGGGAGTTCTAAAGTATTAGAGG
ATCAAAAGATCAGCAGTGGTACAGATGGAAGTTCTCAATTATCAGAAGAAATCACCCAAGGCCAAGGTACAAATGAGACTTGGGAAGTGAT
AGAAAATGTGAGCCATGGTTCACATGTGATTTCTCAAATACCAGAAGAGGAAAGTAAAGATGGTTCAGATGAGACTTCTCAAGTATCAAAAG
AAGAAACCATTGAGTGCTCAAATTGGTTATCTCCAGTAACGGAAGAAGAAATCCATCGAGGTTCGGATGATGTGGACTACATAACAAACCAT
CTTCATGAGCTACTTAATGGTATGAAAGTGCTCATTGTCTTAGATGGCCTATGGGAGGAGGATGCCGATGAGTTGGAGCGGTTGAAGCGTATG
TTGGGAGGAGGTACTAAAGGGAGTAAGGTGACTGTGATCGTAACCACATGCGACGAAGGCATCGCAAGAAAAGTTTGTACCGTTACACCAC
ACAAGCTAAATCCTTTGATGGATGACATGTGCTGGACAATAATCAAGCAACAAAGTGACTTCGAACACAGACCCTACTCTCACAAACAAGTG
TTAGAGCGGATTGGTCTGCAGATTGCAAGTAAGTGTGGGGGTCTACCATTAGCTGCTCAGGAACTTGGGCGCAGTCTACCCAATCAAGATTCT
AGAATTTGGGCAGAGAAAATGAAAGGAGATATCTGGAGTAATATGAGAAGCAACAAGGATGTTATTCAGTCTTTGGTGTTAAGCTACAGAAG
TATGCCACCAGATTTGAGATTATGCTTTGCTTATTGTGCCATCTTTCAGCAGGGTTACATTACAGTCAAAGATGACATCATTCACCAATGGATT
GCCCTCGGTCTTGTTGAACGATCCAACACTCTCTCTGTCACACAGGTAGCTGAGGAGTATATTAGGAGGCTTCTGGACATATCATTCCTTCAAA
CTGCAGAGTCGGCAGCTTCGGTGAGTTGCTAGATACTGCTACTAATTAAGACTTCTCCTCACATTCTGTTGTTCCACAAAAAGATGATACATGC
CTCGATATAAAGAGTCCAGGAGGTTTGTTGTTGTGTGTATGTAAGAATACAAATCTAACTAGAGTTCTACTATTCACAAAAAAAAAAGAGTA
AGTTCTATTGCACGTCAGTTCAGATAACTAAAGTATAACCAAATACCGAAAGACCATGCTATTATAGGCAAACACGTAAATTCATAGTCACA
CATATGCATGAACCTAAAAGAAGTATATTTGGTTTTGGTAATTCATTCCATTGAAGGTCAAGTTATAGCTAGAAAATGTTTGAAGACTGCTATT
AGGCAAAACTCATGTGTAATATATCTAGCACATCTGTCTTTTAAGTATTTCATATTGCAGTTTTGGCGTAAGAAAAATAAGAAAAGTTATACA
CCGTCTCCAAGGAAAAATGGACTTTTAAAAAACTTGAACCAAAGGAAATTATAATGTGAAAATTAAATGGTAATTCTTTTTAAAAACTTGGA
AAATTTGAAGTATGGAAGAGTTTAAAAACAAAGGAAACGATGCCAGCGGTTTGAGGATTTGTACATATTGTGCAATGTAAATCATAGAGTAA
ATGAATGAAAATTATTTTAGAAGAAAAACAGTTGAATGAATTAAATTACGGAAGTGTCTAGAACCAAGGAGAAGTGTTTATGTTTTGAGGTTT
GTGCATATTATATAATGTGCTTAAGTCCAACCATGGAATCTTTTGGTATATGTGGCAAAAATTTGTGTAATGAAAAGGATATCCAATGACCGA
CAAGGATTTGCAACCTATCTACCATTGGATCAGCTTCATCCAATAGCCAATTCTCCTGCATGTGCGGCAGTACCTATTTGGATCCACTTAGTCC
TTTAGCCTGAAATTGAACGAACGGAGCAAAAATAAACAAAAAATGACAATGAAGAGATAAAACTTAAAAATGAAGGGACATAATTTTCGTA
ACTTGAGTAAAATTACAAATATAATGTGAGGAGTGCAAATACCTGGCACTAAACTTCCACTCTCTTCATATGTATAATACTAGACAGTGCGTC
TTCTGAGTTCAAAAATCAAACAAGATTATTAAAAAAATCTAGTTGACTTACAATTAGCAAAAGCGGATGAATAAATGAACGGGACATAATGC
AATGCAAGTACAGCTTATGCATTTCTGGCTTTGTTGCAGACTAGCGGAAATGGTGATACGGGTGCAATCTTGTTGACTATGCACGAAATGGTG
CACGCCCTCTCAAGATCACTTACTGGCGAACTCATTGTTCTGGACGATAGAATGGGTACATCTGATCAGAATAACTGTTGCCGCTATGCACTG
CTTACCGATCTTGGCGCCAAATTATCCAGTAGATTGCCTAACCAGTTAAGGGCGCTACATTGTGTGGGCTATAGCAAAATAAACGTCGGCGAT
GATGCATTTTCATTTACCAGGTGCCTGCGCGTCTTGCATCTAAACGAGTGCTCCATGCACAAGCTACCAGATTCTGTCTGTCAACTGAGGCAGT
TGGGTTATCTTAATTTATCAGGTTGCTCTTGGCTAATGACACTACCAGAATCATTTGGGGGTCTATTAAACCTACGGTATATCAATTTATCACGC
TGCTCTCGGCTAATGACTTTGCCAGAATCATTTGGGGCTCTATTAAACCTACAGTATATCAATTTATCAGGCTGCTCTCAGCTAATGACTCTGC
CAGAATCATTTGGGGGTCTATTAAACCTATGGTATATCAATTTATCACGCTGCAATAGACTTGCAGAACTGCCGGAATCATTCGGAAAGCTAA
AAAGTTTGACTTATCTGGACTTATCATTCTGGTGCTGTTGTGAAGGGATCCGGGTTCTTGGTGGCCTTACCAGTCTCGAGCATTTGAACTTGTCA
CACCCGTGCTGCTATCTTGCTCAACACCGGCCACATCTGGAAGGGCTAAACGAAGTGTTGTGCAAGCTCACCAATCTCCAGTACTTGAACTTA
TCTATGTGTCTGAATCCAATATTTTGCTACCGGTCAGAACAGCAATGTCGTGAATACATACAATCGTGCACTAGTGGCCTTTCCAATCTGCAGC
ATCTGGACTTGTCTCATAACATATTTCTTGATGGTCTACCTGAAAGTCTACGTGAGCTCCAGAAGCTGCATACACTGGATCTCACAGGCTGCAT
TAGACTGGAAAGCGTAGCCAATGAAAGCGACTCTTTGAAGGTGATAGTTATTAGGAATTGCCGGGGTCTGGAAAGTTGCTCTTTTGTGGTTCG
CAGTGATGACAGAAGACTCAGCAGCAACCTCGCTCAGCTCGAGAATGTAAACTGCAAAGTTCTTAAGATAACTTGTCTTGAGAAGGTAAAGT
CTAGAGAGGAAGCCCGAAGGATAAGGCTAGTGGAGAAAAGAAATATTGAAAAGTTGACACTTGGTTGGATTGTGGGCTCTCATGGATCTGTG
GAGGAAGATGCTAATTTGTTAGGAGAACTTAAGCCACCACATGATCTGCAGCGCCTTAAGCTGCACGGCTATCGGGCCACATGCCTCCCGGC
CTGGATGTTGAGCATCTCTTCCTATCTCCCTAATCTTACGAAGGTCACGATGGAGGACTTCCTGAGGTGCAGGAGGCTGCCACCATTCGCTCA
ATTACCAAACCTACAAACTCTGGTTCTCAGAAGGATGTCCAGCATCACAAGAATCGATGCTGGTGAGCTCAGTGGCGATGACACAGCAGCAG
CTGAACTCAAACCACTCTCCAACTTTACCATTGAAGATATGGAAAACCTGGAAGAGTTCAACAGCACATATTACAGTGCTAGCGGTGAGCGG
GTCATGTTCCGCACCATTGATAAATTGGTGATGCGTAAATGCTCCAAGTTGAGTTTTCGGTCTCTCCCTCCTAGAGCTCGGAGCTTAGAGATAT
CAGACTGCGCCCAAGTGCTGTCTTCTTCGAGGTTACTGCTATCAGATTCCACCGAAGTGATTGCATTGCAGAGGACTGCAGGAGGTGCTCCAG
TAGCCGAACTGGTAATCCACGGCAGCATAGCTGACTGGAGTTTGTTTCGCTACCTTGCTGGCCTCCAGACGCTGTCTATCTCTCAATGCGAGGG
CATGACCTCACTGCCACAAGACTTGGATCACCTCAACTCTCTCCAAGACTTGAAGATTGTGCGTTGCAAACAGCTGAAGTCATTTCCAGACAG

CATGAAGCACCTCACCTCCCTCCACTCGATTCATTTGGCTGAGTGCGACAGCATCAAAAAACTTCCCACATGGTTCGGATGCTTCACCTCTCTT
CAGGTACTCTACATTCGTAAGTGCTGTGCCCTTAAATATTTGCCACGGGACATAAAAGGACTCGACAACCTCAAGAATCTGCAAATTATCGAA
TGTCCAAGATTGGAGAACTGGTGTAAGGAGAACAAGAAGAAGTTGGGACGCATTCAACCAACATACGGGTAAGCACCTTCGACCATAGTGC
TCGTTCATTCCACATTCAGGATTACTGTTGCAGACTATTTTACCAATCCATTCATCCATTGCTAAATACTAGATGGCATTTTAACAGTGCCGGAT
CTAGTGGGGATGCCACAGTACCCCCCAAAAAAATGCAAAGTAGTATAGGTATACCATCTTAATATTAATTGTGTAACAATTTAGTGCTATTTC
AGACTATTTAGGCTAAATTATACTCCCTCCGTCCCAAAATTCTTGTCTTAAATTTGTCTAAATACGGATGTATCTAGTTACATTTTAGTGTTAGA
TACATCCGTATCTAAACAAATGTAAGACAAGAATTTTGGGACGGAGGGAATACTGTTTTAGTGTAAATTTTCTATGTTTTGTACAGAAGTATTG
AAGTTTGGCTCCCCGGTACTTAGACTCTAGGTCTGCCACTGCATTTTAACCATGGCACTCTCTTCTGATGCAAGTTTCATCATTATACCAAGTGT
GTTAGTAAAAAGTGGCAACTGTATTGAATCATTTTGATCTCAATAGGTTACTTTTGGGATGTTTCTCCTGATGTCATTTTGAATCATCTTGATCTC
AATAAATTTCATTTGGGATATATGACCACATCCAGAATACATCATCTTAGCTAGCATGGTGTTGCCTTACATCTAATATGTTACTCCACTCGAT
TTTCAGGTGCACTTCCATTTTCTCAGAAGAGGATCCGAATGCTTGTTCTGTAATGTAA

(B)

>ZY1-*CNL5*

ATGGAGCTGGTGGTGGGAGCTTCGGAGGCGACAATGAAGTCCGTCATGGGGAAGCTGGGCGGCCTCCTCGCCCAGGAGTACACCCTGATCCG
GGGCGTCAACGGCGACCTCCAGTACATCAACGACGAGCTGGCCACCATGCAGTCCTTCCTCCGGGACCTCACTGCCGTCGGCGCCGGCCGAG
CCCAGAGCCACGGCCACCGGACGAAGGACTGGATGAAGCAGATCCGCGACATCACCTATGATATCGAGGATTGCATCGACGACTCCGCCCA
TCGCCTCCACGGCCTGCGCTCCGACATGTGCTGCTACTACTTCGCCAACAGCGTCTACGAGGTCCTCACCTGGTGGCCTCGCCGCGACGTCGC
CACCAAGATCTCCGTTCTCAAGATGCGGGCGCAGCAGATCGGGGAGCGGCGGGAGAGGTACGGGGTCAACAACCCGGAGGCCGCCGGTGG
ATCTGAGGGTTCCCATGCCGGAAACGGCACTACTGCCGGATTCGACGCCGCCGACAACCAGGAGGCGAGCCTTGAGCTCGTCGCCATGAAGG
ATCCGGTGGGGGTGGAGGACCACATGAAAGAGCTGGAGGAGTGGCTGACTAACGACAAGAACACCTCCGGCGTGCTGTATGTTGTTGGGTTT
GGAGGGGTGGGGAAGACCACCATTGCTACCGCCTTGTACCGGAAATTTGGGGACCAATTCGACCACCGCGCCATGGTCAACGTGTCTCAGAG
CTCCGATATCAACGCTGTCCTGACCAACATCAAGAATCAAGTCATGCCGCAGTCCAGCGGCCAAACACAGCAAGGCGCCGGAGGCGTGCTC
GGCGGGCTCAAGCGAGGTACCTCGGCATTGGCGGCCAAGTGCTGCAGCGCCGGCGCCTCAGAGGAAACGCGTGGTGAGACGAAGCTGGATC
GGCTCAAGAAAGAGCTCACGGGACACTTTGACAACAGCAGGTACCTTGCACACATCGGACCCTTATTGCTTTCTCTGATTAATGCTTAATGAG
TACAATCCTGATTATGCTGATATAGCAGGATGTGCTTAATTTGTCTTTTATTACTCATGACATAGCAAGCATATATATCATTTTGGGACATGAAT
TATATACATTTACATTTCATTCATTGCTTACTAGGAAAAACAACATGTTTTGGGCCATGGAATTTGGTCCTCATCTATTTTCGTCCTAAATGGAT
GCCATGTGTCAAAAGAAGCCACGGCGCAACCATTTTTTTTTTCCATTCGATGTAAAATGGATCGGATTCCAGTCAGAAATAATGGAACAACAG
ATTGGGTTCCACTATCAGAGATAACGGAACAACAAACAAAATGAAAAAGCATCCTGGTGCAAAACGAAACTACAAAACAGGGTTCAAAGC
GCTAACAACCGCGTGCACCGCTAACAGCCAGATAAACTAAAGTTCTAAAAGGCAACCAATAGTACAGATATTACATAACCAAAACAACCTC
ATTTTTCAGCAACCCTTTTCCCTCCTCTAATATCCAGCTCAATTTGCTTCATCCATGGCTTCATCAAATCGACCATATTGTTCTCCTTTTTGTTCCC
CTCTCATGCTCCAGACAGTGCTCCGCGCCCTATTCGAGCTCCTTGCTGACAGCGCTCCTCTATGTGTGATTTTCTTTTTTTCCTTTTCCTTATTGGC
TTTGTGCATCCAAGTTGTGCATAGGCTGGGTGTGCACTCTCTGTGTTTTATCCACTTGATGTGACATTATGAGTCAATAAAGAGCGCACTTCACT
GGAAAAAAAAAACATACCTTCAGTTCCATGACTGCATAACTTCTTGTTTTAGCTGATGAAATTCATGATCACTTGGTGGGAATCCCTGCCTATG
CAATTATGTTTCTTTTCAATCTTATTCTGTTCCTCAGCCCACATCATTTGATCCAGATAAAATGGGAGGAAAGTAGTACTGTAAACATGAACTG
TACAACCATTTAGTTCTGATAAAGGAGTTATTTATTGCTGCTTACTCACTCTGCTCCATTTTTTTGAAAACTCAATCTCTGCTCCCATTTTTGGTT
TCTTTTCTTGCAGTTACTTGGTCTTGGTTGATGATGTTTGGTCTGCAGCAACGTGGGACCAGATCAGAAAGTCACTTCCGACAAGTAAGAAGCA
CACCAGAATAATAGTTACTACACGGTTTCAAGCTGTTGCTACAAGACATAGAGGGGAAGAAGGTGATCATACTCGCAAAGTTTCCCCTCTTG
GCATTGTAGAATCTGAGAGGCTATTTAAGCAGGCCTTCTCTGAATCCAAAGGTAGTGAGGTTGAAAAGGTTCCAATGGAAGTCTGGAATATGT
GCGGGGGGCTGCCATTGGCAATAGTTATCATGGCTGGTCATGTGGCCTGCAACCCAAACAAATCAGCGGATGAATGGTTGAAGGTCTGCAAA
TCTCTATTTCCAGAGTCAGGGAAGGATCATGGGAAAGGTGGGAGAGACCTTACCCAGGAAGAAGTTGGTAGGATTGTAAGTCATTGCTACAA
TGACATGCCTGCTGACATCAAGACTTGTTCTCTGTATTTGAGCATATTTCCAAAGGGCCAGAAAATCAGCAGGAAGCGTTTGACAAGGCGATG
GATAGCTGAAGCTTTCGTTGCTGAGAAGCAGGGCTTGAGTGTGGAGGACGTTGCAGAGACATATTTCAATCATCTCATAAGAAGGAAGATCA
TCCGACCGGTGGAGCACAGCAGCAATGGGAAGGTGAAGAAATGCATAGTCCACGATATGGTTCTTGAACACATTGTAGCGAAGGCAAGTGA
AGAGAATTTCATCACTGTGATTGGTGGTAACTGGCTTATGCAGCTACCTAGCAGCAAGGTCCGCCGGCTGTCCCTTCAAGAAAGTGACTCGAA
ACGTGCAAATGATACGGAGAAGATGAACCTGTCTCATGTCCGGTCACTGACCATGTTTGGGAGCTTGAACCAACTGCCTTCCCATTCATTCAA
GTTTGGAATTGTACAAGTCCTGGATCTTGAAGGCTGCATGGGTTTCAAAGCGCATCATGCAGAGGAGATATGCAAGATGCTTCTTCTCAAGTA
TCTCAGTCTCCGAAGAAGTGGCACTAAACAGCTTCCAAAAGCAATTGGAAAGCTTGAGAACCTCGAGACTCTGGACATTAGAGAGACAAGT
ATTGTTGAGTTGCCTAAAACCGTATGCCAGCTTGAACGGCTGGTGAACATACTTGGTGGGGATAAAAGAACAAGAAGGGCATTGAAGCTTCC
TCAAGAGTTGAATAAGAAGAAGAAGATGAAGGCCTTGCGCATACTGTCAGGGATTGAGATTGTTGGGGGATTGGCGGACCTTCATCATCTGA
CTGAGCTGAGGAAGCTTGCCATCTACAAGCTCAGCACCATGAGTGACGATCCAAGTTTCAAAGATTTAAGCTCCTCTATCGAGTACCTTGGGG
GCTACTCTCTGAACACCCTCATAATTGACGACGAGTCATCCAAGTTTATTAATTCACTGGATGACCTGTCATCACCACCAAAGTTCCTTGTTGC
CCTTGAGTTATCTGGCAAGATGGTTCAGCTTCCCAGCTGGATCACACAACTCAGTGCTCTCACCAAGCTAACCCTTTCAGTAACAGCTCTCCGG
ACAGATAACTTGCTGCTCCTTAGCAATCTAGATGCACTGTTCTCCCTCACATTCTCATTCAGGGCAGAAAAGCAGGATTCAGAGACACTTACC
ATTCTTGTTGAAAACAAGTTGTCATCTGACGGGGAGATCACAGTCCCAGATGCTGGTTTTAAGAGCCTTAAGCTTCTTCGCTTCTTTGCTCCTCT
CTTGCCTGTATTGAGTTTTTCAGAGAATTCCATGCCAGAGCTGGAAAGGCTAGAGCTGCGGTTCAGCATGCTGGAGGGGCTTTATGGCGTGGA
ATACCTTGCAGGACTCAAGGTGGTGCACCTGACATTGGAGGACAAAGAAGGTGAACATATGACGAAAGAGGTGCAGCGCGAGGTGGAGAG
AGCAGTGAAGAGGACCGATGGCAAGGCACCCAAGATAATCCTCGATCAATGA

Supplementary Data S1. Sequence analysis of known *Pm* genes in three wheat accessions (A) ZY1-*CNL1* (B) ZY1-*CNL5*.

Seed availability statement: The seeds of the wheat accessions used in this study are available from the corresponding author upon reasonable request.

References

1. Hewitt, T.; Müller, M.C.; Molnár, I.; Mascher, M.; Holušová, K.; Šimková, H.; Kunz, L.; Zhang, J.; Li, J.; Bhatt, D.; et al. A highly differentiated region of wheat chromosome 7AL encodes a *Pm1a* immune receptor that recognizes its corresponding *AvrPm1a* effector from *Blumeria graminis*. *New Phytol.* 2021, 229, 2812-2826. https://doi.org/10.1111/nph.17075

2. Jin, Y.; Shi, F.; Liu, W.; Fu, X.; Gu, T.; Han, G.; Shi, Z.; Sheng, Y.; Xu, H.; Li, L.; et al. Identification of resistant germplasm and detection of genes for resistance to powdery mildew and leaf rust from 2,978 wheat accessions. *Plant Dis.* 2021, 105, 3900-3908. https://doi.org/10.1094/PDIS-03-21-0532-RE

3. Sánchez-Martín, J.; Steuernagel, B.; Ghosh, S.; Herren, G.; Hurni, S.; Adamski, N.; Vrána, J.; Kubaláková, M.; Krattinger, S.G.; Wicker, T.; et al. Rapid gene isolation in barley and wheat by mutant chromosome sequencing. *Genome Biol.* 2016, 17, 221. https://doi.org/10.1186/s13059-016-1082-1

4. Yu, Z.; Xiao, L.; Su, F.; Liu, W.; Luo, F.; Han, R.; Mu, Y.; Zhang, W.; Wu, L.; Liang, X.; et al. Mining of wheat *Pm2* alleles for goal-oriented marker-assisted breeding. *Front Plant Sci.* 2022, 13, 912589. https://doi.org/10.3389/fpls.2022.912589

5. Singh, S.P.; Hurni, S.; Ruinelli, M.; Brunner, S.; Sanchez-Martin, J.; Krukowski, P.; Peditto, D.; Buchmann, G.; Zbinden, H.; Keller, B. Evolutionary divergence of the rye *Pm17* and *Pm8* resistance genes reveals ancient diversity. *Plant Mol Biol.* 2018, 98, 249-260. https://doi.org/10.1007/s11103-018-0780-3

6. Zhu, S.; Liu, C.; Gong, S.; Chen, Z.; Chen, R.; Liu, T.; Liu, R.; Du, H.; Guo, R.; Li, G.; et al. Orthologous genes *Pm12* and *Pm21* from two wild relatives of wheat show evolutionary conservation but divergent powdery mildew resistance. *Plant Commun.* 2023, 4. https://doi.org/10.1016/j.xplc.2022.100472

7. He, H.; Zhu, S.; Zhao, R.; Jiang, Z.; Ji, Y.; Ji, J.; Qiu, D.; Li, H.; Bie, T. *Pm21*, encoding a typical CC-NBS-LRR protein, confers broad-spectrum resistance to wheat powdery mildew disease. *Mol Plant.* 2018, 11, 879-882. https://doi.org/10.1016/j.molp.2018.03.004

8. Xing, L.; Hu, P.; Liu, J.; Witek, K.; Zhou, S.; Xu, J.; Zhou, W.; Gao, L.; Huang, Z.; Zhang, R.; et al. *Pm21* from *Haynaldia villosa* encodes a CC-NBS-LRR protein conferring powdery mildew resistance in wheat. *Mol Plant.* 2018, 11, 874-878. https://doi.org/10.1016/j.molp.2018.02.013

9. Li, M.; Dong, L.; Li, B.; Wang, Z.; Xie, J.; Qiu, D.; Li, Y.; Shi, W.; Yang, L.; Wu, Q.; et al. A CNL protein in wild emmer wheat confers powdery mildew resistance. *New Phytol.* 2020, 228, 1027-1037. https://doi.org/10.1111/nph.16761

10. Lu, C.; Du, J.; Chen, H.; Gong, S.; Jin, Y.; Meng, X.; Zhang, T.; Fu, B.; Molnár, I.; Holušová, K.; et al. Wheat *Pm55* alleles exhibit distinct interactions with an inhibitor to cause different powdery mildew resistance. *Nat Commun.* 2024, 15, 503. https://doi.org/10.1038/s41467-024-44796-0

11. Wu, Q.; Chen, Y.; Li, B.; Li, J.; Zhang, P.; Xie, J.; Zhang, H.; Guo, G.; Lu, P.; Li, M.; et al. Functional characterization of powdery mildew resistance gene *MlIW172*, a new *Pm60* allele and its allelic variation in wild emmer wheat. *J Genet Genomics.* 2022, 49, 787-795. https://doi.org/10.1016/j.jgg.2022.01.010

12. Wu, Q.; Zhao, F.; Chen, Y.; Zhang, P.; Zhang, H.; Guo, G.; Xie, J.; Dong, L.; Lu, P.; Li, M.; et al. Bulked segregant CGT-Seq-facilitated map-based cloning of a powdery mildew resistance gene originating from wild emmer wheat (*Triticum dicoccoides*). *Plant Biotechnol J.* 2021, 19, 1288. https://doi.org/10.1111/pbi.13609

13. Li, Y.; Wei, Z.Z.; Fatiukha, A.; Jaiwar, S.; Wang, H.; Hasan, S.; Liu, Z.; Sela, H.; Krugman, T.; Fahima, T. *TdPm60* identified in wild emmer wheat is an ortholog of *Pm60* and constitutes a strong candidate for *PmG16* powdery mildew resistance. *Theor Appl Genet.* 2021, 134, 2777-2793. https://doi.org/10.1007/s00122-021-03858-3

14. Li, Y.; Wei, Z.Z.; Sela, H.; Govta, L.; Klymiuk, V.; Roychowdhury, R.; Chawla, H.S.; Ens, J.; Wiebe, K.; Bocharova, V.; et al. Dissection of a rapidly evolving wheat resistance gene cluster by long-read genome sequencing accelerated the cloning of *Pm69*. *Plant Commun.* 2024, 5. https://doi.org/10.1016/j.xplc.2023.100646

15. Han, G.; Liu, H.; Zhu, S.; Gu, T.; Cao, L.; Yan, H.; Jin, Y.; Wang, J.; Liu, S.; Zhou, Y.; et al. Two functional CC-NBS-LRR proteins from rye chromosome 6RS confer differential age-related powdery mildew resistance to wheat. *Plant Biotechnol J.* 2024, 22, 66-81. https://doi.org/10.1111/pbi.14165

16. He, H.; Wang, J.; Liang, J.; Zhang, Q.; Xue, M.; Chen, Z.; Tang, Q.; Chen, X.; Zhu, S.; Wang, Y. An integrated pipeline facilitates fast cloning of a new powdery mildew resistance gene from the wheat wild relative *Aegilops umbellulata*. *Plant Commun.* 2024, 5. https://doi.org/10.1016/j.xplc.2024.101070

17. Ma, C.; Tian, X.; Dong, Z.; Li, H.; Chen, X.; Liu, W.; Yin, G.; Ma, S.; Zhang, L.; Cao, A.; et al. An *Aegilops longissima* NLR protein with integrated CC-BED module mediates resistance to wheat powdery mildew. *Nat Commun.* 2024, 15, 8281. https://doi.org/10.1038/s41467-024-52670-2

18. Xie, J.; Guo, G.; Wang, Y.; Hu, T.; Wang, L.; Li, J.; Qiu, D.; Li, Y.; Wu, Q.; Lu, P.; et al. A rare single nucleotide variant in *Pm5e* confers powdery mildew resistance in common wheat. *New Phytol.* 2020, 228, 1011-1026. https://doi.org/10.1111/nph.16762

19. Zhu, K.; Li, M.; Dong, L.; Zhang, H.; Zhang, D.; Lu, P.; Wu, Q.; Xie, J.; Chen, Y.; Guo, G.; et al. An atypical NLR pair *TdCNL1*/*TdCNL5* from wild emmer confers powdery mildew resistance in wheat. *Nat Genet.* 2025, 57, 1553-1562. https://doi.org/10.1038/s41588-025-02208-z

20. Sánchez-Martín, J.; Widrig, V.; Herren, G.; Wicker, T.; Zbinden, H.; Gronnier, J.; Spörri, L.; Praz, C.R.; Heuberger, M.; Kolodziej, M.C.; et al. Wheat *Pm4* resistance to powdery mildew is controlled by alternative splice variants encoding chimeric proteins. *Nat Plants.* 2021, 7, 327-341. https://doi.org/10.1038/s41477-021-00869-2

21. Li, H.; Men, W.; Ma, C.; Liu, Q.; Dong, Z.; Tian, X.; Wang, C.; Liu, C.; Gill, H.S.; Ma, P.; et al. Wheat powdery mildew resistance gene *Pm13* encodes a mixed lineage kinase domain-like protein. *Nat Commun.* 2024, 15, 2449. https://doi.org/10.1038/s41467-024-46814-7

22. He, H.; Chen, Z.; Fan, R.; Zhang, J.; Zhu, S.; Wang, J.; Zhang, Q.; Gao, A.; Gong, S.; Zhang, L.; et al. A kinase fusion protein from *Aegilops longissima* confers resistance to wheat powdery mildew. *Nat Commun.* 2024, 15, 6512. https://doi.org/10.1038/s41467-024-50909-6

23. Lu, P.; Guo, L.; Wang, Z.; Li, B.; Li, J.; Li, Y.; Qiu, D.; Shi, W.; Yang, L.; Wang, N.; et al. A rare gain of function mutation in a wheat tandem kinase confers resistance to powdery mildew. *Nat Commun.* 2020, 11, 680. https://doi.org/10.1038/s41467-020-14294-0

24. Gaurav, K.; Arora, S.; Silva, P.; Sánchez-Martín, J.; Horsnell, R.; Gao, L.; Brar, G.S.; Widrig, V.; John Raupp, W.; Singh, N.; et al. Population genomic analysis of *Aegilops tauschii* identifies targets for bread wheat improvement. *Nat Biotechnol.* 2022, 40, 422-431. https://doi.org/10.1038/s41587-021-01058-4

25. Li, M.; Zhang, H.; Xiao, H.; Zhu, K.; Shi, W.; Zhang, D.; Wang, Y.; Yang, L.; Wu, Q.; Xie, J.; et al. A membrane associated tandem kinase from wild emmer wheat confers broad-spectrum resistance to powdery mildew. *Nat Commun.* 2024, 15, 3124. https://doi.org/10.1038/s41467-024-47497-w

26. Zhao, Y.; Dong, Z.; Miao, J.; Liu, Q.; Ma, C.; Tian, X.; He, J.; Bi, H.; Yao, W.; Li, T.; et al. *Pm57* from *Aegilops searsii* encodes a tandem kinase protein and confers wheat powdery mildew resistance. *Nat Commun.* 2024, 15, 4796. https://doi.org/10.1038/s41467-024-49257-2

27. Lagudah, E.S.; Krattinger, S.G.; Herrera-Foessel, S.; Singh, R.P.; Huerta-Espino, J.; Spielmeyer, W.; Brown-Guedira, G.; Selter, L.L.; Keller, B. Gene-specific markers for the wheat gene *Lr34*/*Yr18*/*Pm38* which confers resistance to multiple fungal pathogens. *Theor Appl Genet.* 2009, 119, 889-898. https://doi.org/10.1007/s00122-009-1097-z

28. Moore, J.W.; Herrera-Foessel, S.; Lan, C.; Schnippenkoetter, W.; Ayliffe, M.; Huerta-Espino, J.; Lillemo, M.; Viccars, L.; Milne, R.; Periyannan, S.; et al. A recently evolved hexose transporter variant confers resistance to multiple pathogens in wheat. *Nat. Genet.* 2015, 47, 1494-1498. https://doi.org/10.1038/ng.3439

29. He, H.; Tang, Q.; Zhang, Q.; Zhu, S.; Lv, S.; Bao, Y.; Liang, J.; Wang, J.; Wang, J.; Xu, H.; et al. An NLR pair in the *Pm68* locus confers powdery mildew resistance in durum and common wheat. *Nat Commun.* 2025, 16, 9039. https://doi.org/10.1038/s41467-025-64048-z

30. Yang, Z.; Liu, N.; Xie, X.; Wei, W.; Bai, Y.; Sun, J.; Pan, W.; Yang, J.; Wang, W.; Xie, X.; et al. Two complementary NLRs from wild emmer wheat confer powdery mildew resistance. *Nat Commun.* 2025, 16, 9041. https://doi.org/10.1038/s41467-025-64052-3

31. Zhang, H.; Li, M.; Wang, G.; Zhu, K.; Guo, G.; Fu, H.; Hu, C.; Chu, Z.; Hu, J.; Wu, Q.; et al. Paired NLRs originated from *Triticum dicoccoides* coordinately confer resistance to powdery mildew in wheat. *Nat Commun.* 2025, 16, 9040. https://doi.org/10.1038/s41467-025-64049-y

32. Wang, Z.; Xie, J.; Zhang, H.; Zhang, J.; He, F.; Cui, B.; Dolezel, J.; Cai, X.; Ding, Z.; Luo, Q.; et al. *Pm6* from *Triticum timopheevii* encodes an NLR receptor that directly recognizes *AvrPm6* to confer powdery mildew resistance in wheat. *Mol. Plant*. 2025, 18, 2040-2044. https://doi.org/10.1016/j.molp.2025.10.012

33. Jin, Y.; Li, W.; Li, Y.; Li, D.; Yan, H.; Chen, S.; Han, G.; Xiao, B.; Li, B.; Lu, P.; et al. *Pm37* as a susceptible *Sr22* allele confers resistance to wheat powdery mildew and leaf rust. *Nat Commun*. 2026, 17, 3165. https://doi.org/10.1038/s41467-026-69717-1

34. Wan, W.; Zhao, R.; Zhao, P.; Tang, Q.; Lv, G.; Chen, T.; Wang, L.; Zang, S.; Wu, R.; Wang, Z.; et al. MutExomeSeq accelerates the cloning of *PmNCA6* conferring powdery mildew resistance from *Triticum boeoticum*. *Engineering* 2026. https://doi.org/10.1016/j.eng.2026.02.027

35. Tang, H.; Lyu, B.; Yu, Y.; Wang, L.; Liu, X.; Zheng, X.; Yan, B.; Li, Z.; Zhang, T.; Chen, X.; et al. *PmD479* is an unutilized gene for powdery mildew resistance in common wheat. *Plant Biotechnol J*. 2026. https://doi.org/10.1111/pbi.70704
